# Biophysical and structural characterization of the impacts of MET phosphorylation on tepotinib binding

**DOI:** 10.1016/j.jbc.2023.105328

**Published:** 2023-10-06

**Authors:** Ulrich Grädler, Daniel Schwarz, Ansgar Wegener, Thomas Eichhorn, Tiago M. Bandeiras, Micael C. Freitas, Alfred Lammens, Oleg Ganichkin, Martin Augustin, Stefano Minguzzi, Frank Becker, Jörg Bomke

**Affiliations:** 1 The Healthcare Business of Merck KGaA, Darmstadt, Germany; 2 Merck KGaA, Darmstadt, Germany; 3 iBET, Instituto de Biologia Experimental e Tecnológica, Oeiras, Portugal; 4 Proteros Biostructures GmbH, Planegg, Germany; 5 Intana Bioscience GmbH, Planegg, Germany

**Keywords:** MET, tepotinib, mesenchymal-epithelial transition factor, phosphorylation, surface plasmon resonance (SPR), X-ray crystallography, mass spectrometry (MS), conformational change

## Abstract

The receptor tyrosine kinase MET is activated by hepatocyte growth factor binding, followed by phosphorylation of the intracellular kinase domain (KD) mainly within the activation loop (A-loop) on Y1234 and Y1235. Dysregulation of MET can lead to both tumor growth and metastatic progression of cancer cells. Tepotinib is a highly selective, potent type Ib MET inhibitor and approved for treatment of non–small cell lung cancer harboring *MET*ex14 skipping alterations. Tepotinib binds to the ATP site of unphosphorylated MET with critical π-stacking contacts to Y1230 of the A-loop, resulting in a high residence time. In our study, we combined protein crystallography, biophysical methods (surface plasmon resonance, differential scanning fluorimetry), and mass spectrometry to clarify the impacts of A-loop conformation on tepotinib binding using different recombinant MET KD protein variants. We solved the first crystal structures of MET mutants Y1235D, Y1234E/1235E, and F1200I in complex with tepotinib. Our biophysical and structural data indicated a linkage between reduced residence times for tepotinib and modulation of A-loop conformation either by mutation (Y1235D), by affecting the overall Y1234/Y1235 phosphorylation status (L1195V and F1200I) or by disturbing critical π–stacking interactions with tepotinib (Y1230C). We corroborated these data with target engagement studies by fluorescence cross-correlation spectroscopy using KD constructs in cell lysates or full-length receptors from solubilized cellular membranes as WT or activated mutants (Y1235D and Y1234E/1235E). Collectively, our results provide further insight into the MET A-loop structural determinants that affect the binding of the selective inhibitor tepotinib.

The mesenchymal-epithelial transition factor (MET) belongs to the family of receptor tyrosine kinases and is activated by its cognate high-affinity ligand hepatocyte growth factor (HGF) (1, 2). HGF binding to MET receptor activates several downstream signaling pathways including mitogen-activated protein kinase, PI3K, signal transducer/activator of transcription protein, and NF-κB (3). Dysregulation of MET signaling resulting either from overexpression, mutational activation, or amplification has been observed in many cancer types, including liver and lung cancer (4, 5, 6). Patients with non–small cell lung cancer (NSCLC), carrying a *MET* splicing variant resulting in exon 14 skipping (*MET*ex14 skipping) mutations and impaired receptor breakdown, are responsive to small molecule inhibitors of MET (7). The current understanding of MET activation is based on a recent cryo-EM structure (PDB-ID: 7MO7) revealing an asymmetric 2:2 MET:HGF complex with one HGF molecule bridging two MET extracellular domains and a second HGF molecule stabilizing the active conformation in conjunction with heparin (8). HGF-stimulated MET dimerization induces sequential phosphorylation of the intracellular kinase domain (KD) initially on Y1235 and followed by Y1234, which is involved in an H-bond interaction stabilizing the activation loop (A-loop) within the autoinhibited MET conformation (9, 10). Dual phosphorylation on Y1234 and Y1235 results in structural rearrangement of the A-loop toward the active KD conformation (11). The oncogenic MET mutation Y1235D, which is described for different cancer types such as hereditary papillary renal carcinoma and advanced head and neck squamous cell carcinoma, leads to constitutive kinase activation presumably by destabilizing the autoinhibited KD conformation (10, 12, 13, 14). Other mutations comprise D1228H/N/V or Y1230C/D/H within the A-loop, which are mediating drug resistance of type I MET inhibitors (15, 16, 17). Further mutations outside of the ATP pocket including F1200I/L and L1195V affect potencies of type II MET inhibitors such as cabozantinib (16, 18). First insights into structural impacts by oncogenic MET mutations L1195V, D1228V, Y1230H, and M1250T were gained from recent crystal structures in complex with type I and type II MET inhibitors (19, 20).

Tepotinib (TEPMETKO, MSC2156119J, Table S1) is an oral, highly selective, potent type Ib ATP-competitive MET inhibitor, which has been approved in Japan and the USA for the treatment of adults with advanced or metastatic NSCLC harboring *MET*ex14 skipping alterations (21, 22, 23, 24, 25, 26). The cocrystal structure of tepotinib bound to the KD of WT MET at 1.2 Å resolution (PDB-ID: 4R1V) revealed a U-shaped conformation within the ATP-binding pocket with critical H-bonds of the pyrimidine ring to the hinge region, which is similar to other MET type Ib inhibitors such as capmatinib and savolitinib (27, 28). In this structure, the A-loop is completely defined and protrudes into the ATP-site facilitating π–stacking interactions between the tepotinib pyridazinone ring and the Y1230 sidechain. This WT MET structure in complex with tepotinib adopts an inactive kinase conformation comprising an αC-helix out conformation with both A-loop tyrosines Y1235 and Y1234 in the unphosphorylated state. In this study, we present more detailed biophysical and structural data of tepotinib binding to different conformational states of MET induced either by A-loop phosphorylation (Y1234, Y1235) or by oncogenic mutations. In this regard, we investigated selected mutations located in the A-loop (Y1235D), outside the ATP-site (F1200I, L1195V), and in direct binding interaction with tepotinib (Y1230C). We solved the first crystal structures of MET mutants F1200I, Y1235D, and Y1234E/Y1235E in complex with tepotinib, which also represent the first published crystal structures bearing these MET mutations. Finally, we complemented our biophysical and structural data with target engagement studies using fluorescence cross-correlation spectroscopy (FCCS) in a more physiological environment with KD constructs in cell lysates and full-length receptors embedded in solubilized cellular membranes.

## Results

### Tepotinib binds with a high residence time to unphosphorylated MET

**Figure 1:**
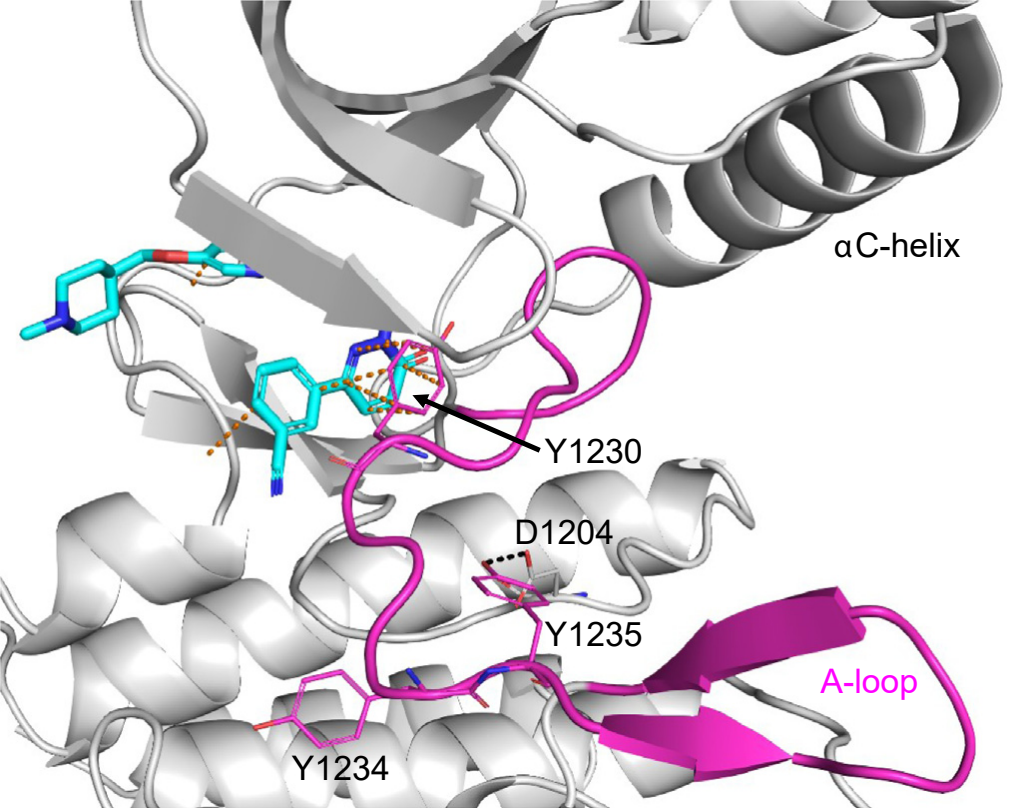
**The crystal structure of unphosphorylated WT MET revealed a buried binding position of tepotinib with the A-loop in the autophosphorylated conformation.** Crystal structure (PDB-ID: 4R1V) of WT MET in complex with tepotinib (*cyan*) revealed critical π–stacking interactions (*dashed orange lines*) between the tepotinib pyridazinone ring and Y1230 of the A-loop (*magenta*), which is oriented toward the ATP pocket involving an H-bond interaction (*dashed black line*) between the unphosphorylated Y1235 and D1204. Electron density indicated no phosphorylation on Y1234, which is oriented toward the solvent. A-loop, activation loop; MET, mesenchymal-epithelial transition factor.

Purified recombinant WT MET KD (28) used in our study is present as a ∼1:1 mixture of unphosphorylated (0P) and mono-phosphorylated (1P) species (Fig. S1) as described before (29). It was not possible to prepare completely unphosphorylated protein material or to separate the 0P and 1P protein species from the mixture for binding studies (data not shown). Thus, we used this protein sample in surface plasmon resonance (SPR) binding experiments and observed a low off-rate binding kinetic (k_d_ <0.0001 s^−1^) for tepotinib corresponding to a residence time of >160 min (Table 1). Further kinetic parameters such as the K_D_ values or the on-rate for tepotinib could not be determined because of technical limitations due to neglectable dissociation of bound tepotinib from the immobilized protein on the SPR surface (Fig. S2*A*). In a biochemical assay using the same recombinant protein and in absence of ATP preincubation, we measured an IC_50_ value of 1.7 nM for tepotinib (Table 2) (30). The slow dissociation kinetics of tepotinib can be explained by the highly buried binding mode within the ATP site observed in the WT MET cocrystal structure (PDB-ID: 4R1V) (28). In this structure, the completely defined A-loop protrudes into the ATP pocket, facilitating π–stacking interactions between the tepotinib pyridazinone ring and Y1230 sidechain, thus blocking dissociation of the bound inhibitor (Fig. 1). The tight interaction of tepotinib to WT MET protein was in line with significant thermal stabilization effects observed in differential scanning fluorimetry (DSF) studies using either SYPRO orange dye (thermal shift assay: ΔT_m_ = +15 °C) or the intrinsic protein fluorescence (nDSF: ΔT_m_ = +18.6 °C) as readouts (Table 2). The WT MET•tepotinib structure showed no additional electron density corresponding to phosphorylation on Y1234 or Y1235, which are either solvent exposed (Y1234) or involved in an H-bond interaction (Y1235) to D1204 (Fig. 1). Modeling suggests that phosphorylation on Y1234, which is present in the 1P form according to our mass spectrometry (MS) results (Fig. S1), would be tolerated without affecting the A-loop conformation in the MET•tepotinib structure because no steric clashes or unfavorable electrostatic interactions are caused. In contrast, phosphorylation on Y1235 would disrupt its H-bond to D1204 and destabilize the A-loop conformation in the WT MET•tepotinib structure. Next, we examined the impacts of Y1234 and Y1235 phosphorylation within the A-loop on tepotinib binding.

**Table 1:** Affinity and kinetic interaction parameters determined by SPR

MET (1051–1349) protein variants	Tepotinib			
	*K*_D_^a^ (nM)	*k*_d_^b^ (s^−1^)	*k*_a_^c^ (M^−1^ s^−1^)	Residence time^d^ (min)
WT (no ATP preincubation)	NA	<0.0001	NA	>160
WT (after ATP preincubation)	5.7	0.0095	1,670,000	1.8
WT mix (species C1)	0.08	0.00012	1,490,000	138
WT mix (species C2)	2.4	0.0064	2,690,000	2.6
Y1234E/Y1235E	2.9	0.0079	2,755,000	2.1
Y1235D	2.3	0.0057	2,500,000	2.9
F1200I (species G1)	0.5	0.0057	11,500,000	2.9
F1200I (species G2)	23.4	0.0208	888,150	0.8
L1195V (species H1)	0.3	0.0009	3,060,000	18.5
L1195V (species H2)	80.3	0.0345	430,000	0.5
Y1230C	1208	0.29	240,000	<0.1

NA, not applicable, ND, not determined.

a Equilibrium dissociation constants (k_d_/k_a_).

b Dissociation rate constants.

c Association rate constants.

d Residence time (RT = 1/(k_d_)). WT mix: WT protein samples prior and after ATP preincubation were mixed in a 1:1 ratio and immobilized for SPR experiments.

**Table 2:** Biophysical and biochemical evaluation of WT and mutant MET proteins

MET (1051–1349) protein variants	Tepotinib			
	TSA		nDSF	Biochemical IC_50_ (nM)
	T_m_ (°C)	ΔT_m_ (°C)^a^	ΔT_m_ (°C)^a^	
WT (no ATP preincubation)	47	+15	+18.6	1.7
WT (after ATP preincubation)	47	+10	+13.5	NT
Y1234E/Y1235E	47	+10	+13.9	NT
Y1235D	48	+11	+14	1.7
F1200I	53	+9	+9.9	232
L1195V	49	+4/10^b^	+8.5/13.4^c^	300
Y1230C	48	+1	+4.3	3900

NT, not tested; TSA, thermal shift assay.

a T_m_ shifts calculated based on main transition.

b Two transitions observed in TSA.

c Two transitions observed in nDSF based on two different T_m_ values for the apo protein.

### MET autophosphorylation significantly reduces the residence time of tepotinib

WT MET KD was autophosphorylated by incubating the protein with ATP/Mg^2+^, and the resulting material was dual-phosphorylated (2P) at Y1234 and Y1235 (Fig. S1) in agreement with earlier reports (29, 31). SPR experiments with the autophosphorylated protein sample (Table 1) indicated that the high-binding affinity (K_D_ = 5.7 nM) of tepotinib was maintained but revealed an increased off-rate (k_d_ = 0.0095 s^−1^) corresponding to a calculated residence time of 1.8 min (Fig. S2*B*). For further dissection of the kinetic parameters corresponding to differently phosphorylated MET protein species, we conducted SPR experiments with a ∼1:1 mixture of WT proteins prior to and after 5 min of ATP/Mg^2+^ incubation. The sensorgrams in the SPR overlay plots were separated into two subpopulations, representing a high affinity (C1) and low affinity (C2) species with distinct K_D_-values and residence times derived from a 1:1 Langmuir binding model (Fig. S2*C*). The dissected kinetic parameters for the high affinity (C1: K_D_ = 0.08 nM, residence time = 138 min) and low affinity (C2: K_D_ = 2.4 nM, residence time = 2.6 min) species corresponded well with values observed with WT protein prior to and after autophosphorylation. Thermal stabilization of the 2P protein species upon tepotinib binding was also significantly reduced (thermal shift assay: ΔT_m_ = +10 °C, nDSF: ΔT_m_ = +13.5 °C, Table 2).

### MET Y1234E/Y1235E crystal structure with tepotinib shows disordered A-loop

**Figure 2:**
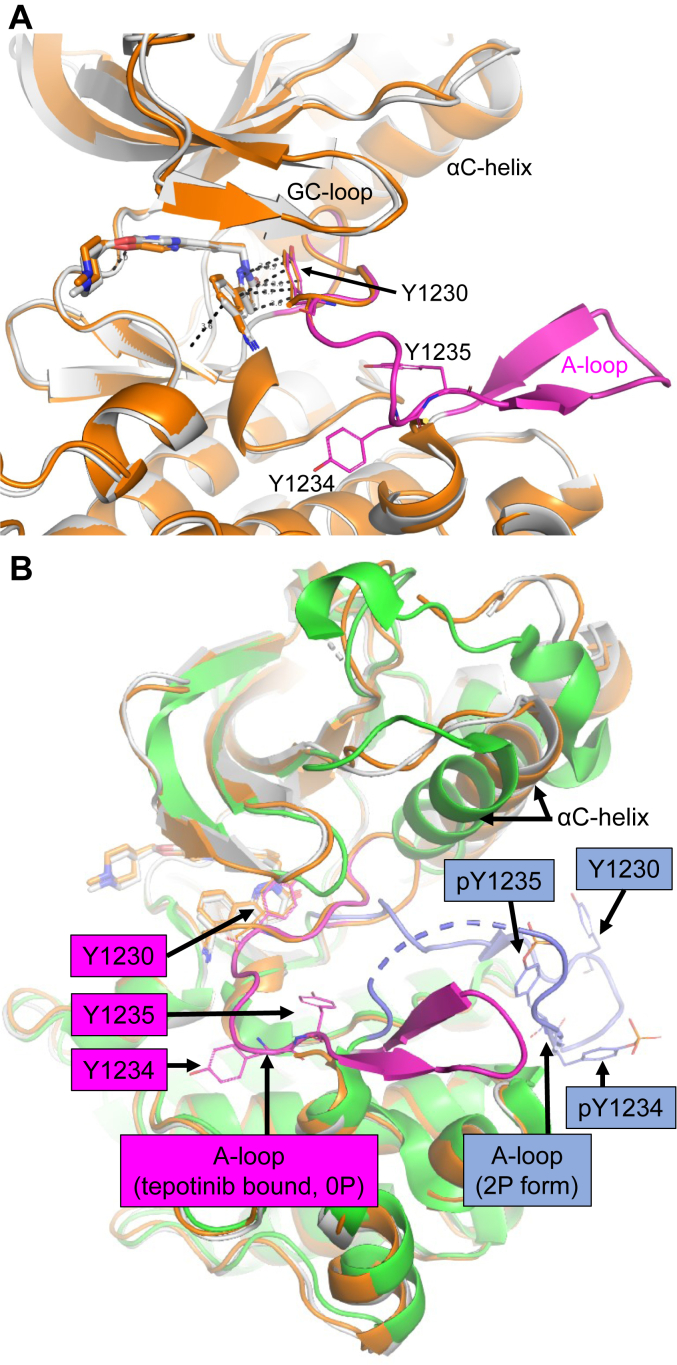
**The crystal structure of the phosphorylation mimicking Y1234E/Y1235E mutant showed an identical tepotinib binding position as in the WT structure and a mainly disordered A-loop.***A*, structural superimposition of MET Y1234E/Y1235E•tepotinib (*orange*, PDB-ID: 8AU3) and WT MET•tepotinib (*gray*, A-loop in *magenta*) revealed identical π–stacking interactions (*dashed black lines*) between the tepotinib pyridazinone ring and Y1230 of the A-loop, which is disordered between residues 1232 to 1244 in the Y1234E/Y1235E structure. *B*, overlay of MET crystal structures of the unphosphorylated WT (*gray*, A-loop in *magenta*, PDB-ID: 4R1V) and Y1234E/Y1235E in complex with tepotinib (*orange*, PDB-ID: 8AU3) and the dual-phosphorylated form (*green*, A-loop in *blue*, PDB-ID: 4IWD) show significantly different A-loop conformations induced by Y1234 and Y1235 phosphorylation. In addition, the tepotinib-bound structure adopts an αC-helix out/DFG-in conformation, while the dual-phosphorylated structure is found in the αC-helix in/DFG-in conformation. A-loop, activation loop; MET, mesenchymal-epithelial transition factor.

We assumed a large impact upon phosphorylation on both Y1234 and Y1235 causing rearrangements of the A-loop and αC-helix conformations towards the active KD form as observed for the dual-phosphorylated MET structure (Fig. S3) (11). Unfortunately, no cocrystals of autophosphorylated MET WT protein with tepotinib could be obtained. Therefore, we generated a phosphomimetic MET Y1234E/Y1235E mutant expressed from insect cells as a surrogate protein for structural studies. SPR studies with MET Y1234E/Y1235E (Table 1) showed a similar binding affinity (K_D_ = 2.9 nM) and off-rate (k_d_ = 0.0079 s^−1^, residence time = 2.1 min) for tepotinib as for the 2P form (Fig. S2*D*). Accordingly, Y1234E/Y1235E thermal shifts for tepotinib were also identical to the 2P form (Table 2). Due to these molecular interaction similarities, we then opted to use the Y1234E/Y1235E mutant as a mimic of the 2P form to enable crystallographic experiments. We solved the crystal structure of MET Y1234E/Y1235E with tepotinib at 2.26 Å resolution (PDB-ID: 8AU3, Table 3), which overlays closely with that of the WT structure (Fig. 2*A*). Although the A-loop was disordered between K1232 and K1244, electron density for Y1230 was still defined indicating π–stacking interactions with tepotinib identical to the WT structure. Overall, the A-loop and αC-helix conformations are similar in the tepotinib-bound MET WT and Y1234E/Y1235E structures, but distinct to the dual-phosphorylated structure (Fig. 2*B*). The enhanced A-loop disorder in the Y1234E/Y1235E structure compared to the WT structure with a fully ordered A-loop might explain the large reduction in residence time for tepotinib observed for the Y1234E/Y1235E mutant.

**Table 3:** Summary of crystallographic information

PDB-ID	MET Y1234E/Y1235E∙tepotinib	MET Y1235D∙tepotinib	MET F1200I∙tepotinib
	8AU3	8AW1	8AU5
Data collection and processing			
Wavelength (Å)	1.000	1.000	1.000
Space group	F2 2 2	P2_1_	P2_1_
Unit cell parameters			
a, b, c (Å)	116.68, 139.12, 240.12	77.83, 43.29, 89.29	38.48, 43.17, 88.79
α, β, γ (°)	90.0, 90.0, 90.0	90.0, 90.6, 90.0	90.0, 91.7, 90.0
Resolution range (Å)	120–2.26	89–2.14	89–2.72
Unique reflections	28,169 (1408)	32,217 (9089)	7637 (1783)
*R(I)*_*sym*_ (%)^a^	8.7 (118.3)	5.6 (44.1)	5.0 (47.2)
Completeness (%)	92.2 (66.6)	96.6 (97.6)	95.0 (96.7)
Redundancy	8.2	2.9	2.9
I/σ (I)	14.9 (1.8)	12.9 (2.8)	14.2 (2.9)
Refinement			
Resolution range (Å)	120–2.26	89–2.14	89–2.72
Reflections used in refinement (work/free)	27,348/822	30,653/1564	6891/746
Final R values for all reflections (work^b^/free^c^) (%)	19.7/24.0	20.8/24.9	23.4/27.8
No. Atoms			
Proteins	4510	4230	2123
Ligands/others^d^	74/20	74	37/13
Water	82	153	3
RMSDs (protein residues)			
Bonds (Å)	0.011	0.011	0.009
Angles (°)	1.57	1.32	1.05
Ramachandran parameters (%)^e^			
Most favoured regions	91.2	92.7	88.4
Additional allowed regions	8.4	6.7	11.1
Generously allowed regions	0.4	0.7	0.4
Disallowed regions	0	0	0
Mean B-factor (Å^2^)	4.0	2.5	1.9

Numbers in parentheses represents the highest resolution shell.

a $R_{sym}=\left(\sum \left|\left(I\;-\;\left\langle I\right\rangle \right)\right|/\sum I\right)\text{,}$ where ⟨I⟩ is the average intensity of multiple measurements.

b $R_{work}=\left(\sum \left|\left(F_{obs}\;-\;F_{calc}\right)\right|/\sum \left|F_{obs}\right|\right)$.

c *R*_*free*_ = *R*_*work*_ based on ∼1000 (at least 5%) of reflections excluded from refinement.

d Ligands/ion are tepotinib and PEG.

e Calculated using PROCHECK.

### Tepotinib adopts an identical binding mode and shows similar binding kinetics toward the oncogenic MET Y1235D mutant as observed for the WT form

**Figure 3:**
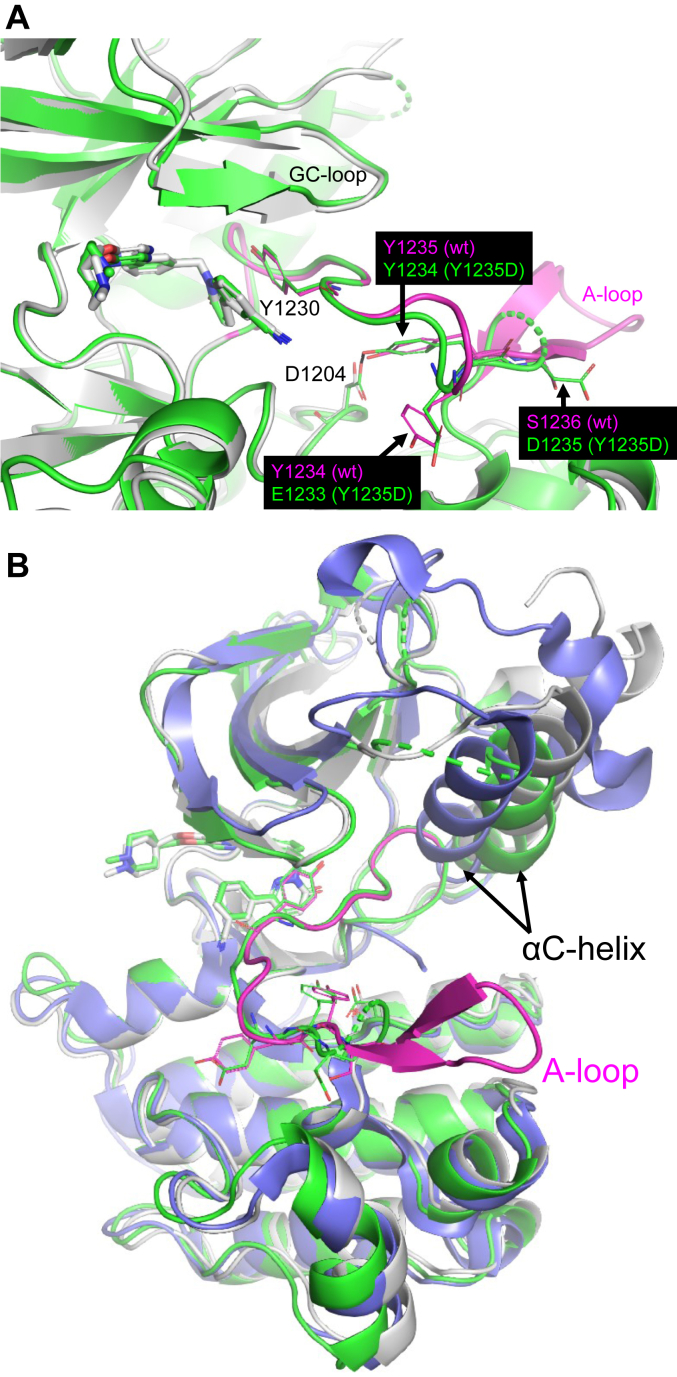
**The crystal structure of the oncogenic MET Y1235D mutant showed an identical binding mode for tepotinib as in the WT involving a positional frame shift of Y1234 and Y1235D.***A*, structural superimposition of MET Y1235D•tepotinib (*green*, PDB-ID: 8AW1) and WT MET•tepotinib (*gray*, A-loop in *magenta*) revealed identical inhibitor and Y1230 positions. The A-loop in the Y1235D structure indicated a frame shift involving E1233, Y1234, and D1235, which respectively adopt the positions of Y1234, Y1235, and S1236 in the WT structure. *B*, overlay of MET Y1235D•tepotinib (*green*, PDB-ID: 8AW1), WT MET•tepotinib (*gray*, A-loop in *magenta*), and the dual-phosphorylated apo form (*blue*: PDB-ID: 3Q6U) showed different αC-helix positions, indicating an inactive KD conformation present in the tepotinib complexes. A-loop, activation loop; KD, kinase domain; MET, mesenchymal-epithelial transition factor.

We next investigated the binding properties of tepotinib towards the oncogenic and activating MET Y1235D mutant to complement our studies with the phosphomimetic Y1234E/Y1235E mutant. Recombinant MET Y1235D protein was obtained from insect cells expression in the nonphosphorylated form as confirmed by MS analysis (Fig. S4). The MET Y1235D mutant revealed nearly identical SPR results (Table 1, Fig. S2*E*) and thermal stability shifts (Table 2) for tepotinib as observed for the WT 2P form and Y1234E/Y1235E mutant. The biochemical potency for tepotinib against the Y1235D mutant was identical to the WT protein and in good agreement with the SPR K_D_ value (Table 2). We solved the crystal structure of MET Y1235D with tepotinib at 2.14 Å resolution (PDB-ID: 8AW1, Table 3), which revealed an identical inhibitor binding mode in an αC-helix out/DFG-in protein conformation as in the WT and Y1234E/Y1235E structures (Fig. 3*A*). Again, some parts of the A-loop were disordered (residues 1236–1243), but electron density for Y1230 indicated identical π–stacking interactions to tepotinib as in the WT structure. Surprisingly, we observed a frame shift in the A-loop of the Y1235D structure involving Y1234, which adopts the position of Y1235 in the WT structure (Fig. 3*A*). Consequently, D1235 in the Y1235D structure is shifted toward the position of S1236 in the WT structure, which is solvent exposed. Overall, the frame shift in the Y1235D structure avoids an opposing positioning of two negatively charged side chains that would result by introducing the Y1235D mutation into the WT structure with Y1235 involved in an H-bond interaction to D1204 (Fig. 1).

### Impacts of MET F1200I, L1195V, and Y1230C mutations on tepotinib binding

**Figure 4:**
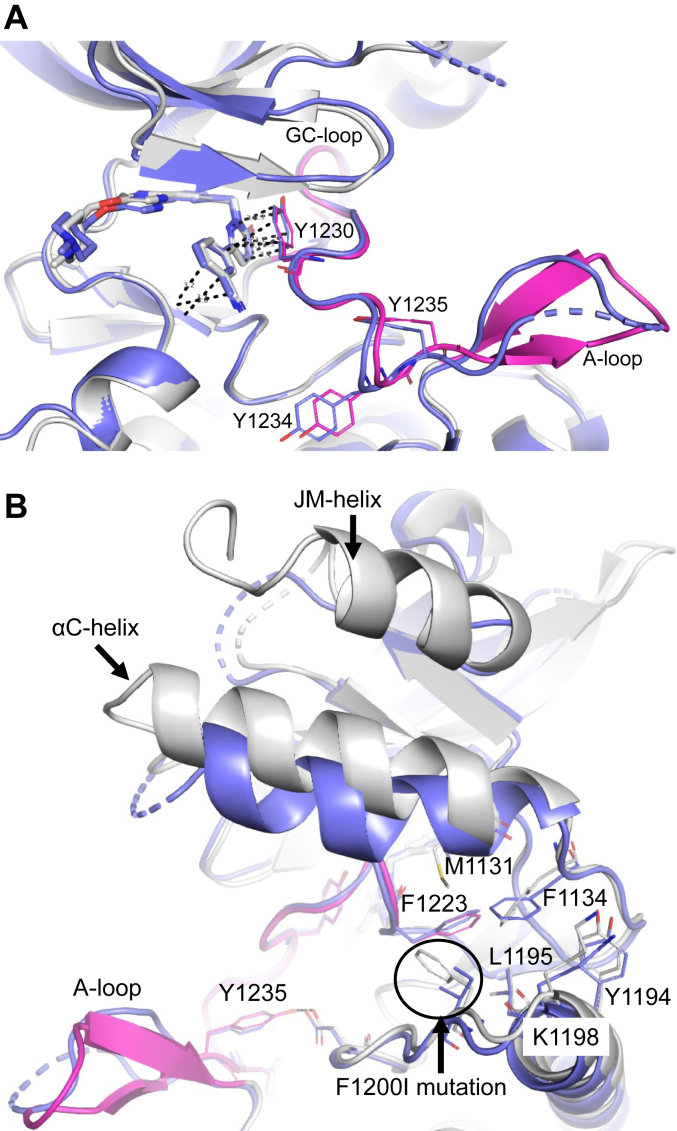
**The F1200I structure showed enhanced A-loop disorder.***A*, overlayed crystal structures of MET F1200I•tepotinib (*blue*, PDB-ID: 8AU5) and WT MET•tepotinib (*gray*, A-loop in *magenta*) showed identical positions of the inhibitor, GC-loop, and A-loop, including Y1230 (π–stacking interactions are represented as *dashed black lines*), Y1234 and Y1235. *B*, the αC-helix is slightly shifted in the MET F1200I•tepotinib (*blue*, PDB-ID: 8AU5) structure compared to the WT MET•tepotinib structure (*gray*, A-loop in *magenta*). The F1200I mutation is situated in a hydrophobic cleft formed by: M1131, F1134, Y1194 (phosphorylation site), L1195 (known as oncogenic mutation position), F1223 (DFG motif). The JM-helix (N1058-V1070) on top of the kinase N-lobe is disordered in the F1200I structure. A-loop, activation loop; MET, mesenchymal-epithelial transition factor.

We completed our biophysical binding studies with selected oncogenic mutants, which are located either distant to the ATP site (F1200I and L1195V) or involved in direct π–stacking interaction with tepotinib (Y1230C) to understand their impact on tepotinib binding. Recombinant F1200I, L1195V, and Y1230C proteins were again obtained from insect cell expression and phosphorylation status analysis revealed a mixture of phosphorylation states for each protein (Figs. S4–S7). In contrast to the WT and Y1230C proteins, which contained mixtures of the 0P and 1P forms, the recombinant F1200I and L1195V protein samples indicated the 2P form as major species present after purification and prior to additional autophosphorylation (Figs. S4–S7). Isolation of dephosphorylated protein or the 2P form to conduct SPR experiments failed (data not shown). In SPR studies using F1200I and L1195V proteins without ATP incubation, we observed the presence of high affinity (F1200I/L1195V: K_D_ = 0.5/0.3 nM, residence time = 2.9/18.5 min) and low affinity species (F1200I/L1195V: K_D_ = 23.4/80.3 nM, residence time = 0.8/0.5 min) for tepotinib binding as expected based on the presence of multiple phosphorylation states (Table 1, Fig. S2). In the biochemical assay with the F1200I and L1195V mutants, we found reduced affinities for tepotinib (IC_50_s∼300 nM, Table 2) in comparison to the WT. DSF experiments with untreated F1200I and L1195V proteins showed similar transitions for tepotinib as seen for the 2P form and Y1234E/Y1235E mutant (Table 2). In contrast, the Y1230C mutant indicated significant reductions of binding affinity (SPR K_D_ = 1208 nM, biochemical IC_50_ = 3900 nM) and residence time (<0.1 min) (Tables 1 and 2, Fig. S2). In addition, thermal shifts for tepotinib against the Y1230C mutant were significantly reduced compared with the WT (Table 2). We solved the crystal structure of MET F1200I with tepotinib at 2.72 Å resolution (PDB-ID: 8AU5, Table 3) and found an identical binding position for tepotinib with π–stacking interactions to Y1230 as in the WT, Y1234E/Y1235E, and Y1235D structures (Fig. 4*A*). The A-loop was nearly completely defined, except for H1238 and D1239, comprising a similar conformation as in the WT structure also regarding Y1234 and Y1235 positions, which are both unphosphorylated (Fig. 4*A*). The A-loop conformations in the unphosphorylated WT and F1200I structures were identical but we noticed enhanced disorder of the A-loops in the Y1234E/Y1235E and Y1235D structures. In comparison with the WT structure, the αC-helix was slightly disordered, and the adjacent JM-helix located above the kinase N-lobe was disordered in the F1200I structure (Fig. 4*B*).

### Crystallographic packing might not impact A-loop conformations observed in MET cocrystal structures with tepotinib

We further analyzed all MET•tepotinib crystal structures with PDBePISA (32) to clarify, if the different A-loop and αC-helix conformations are influenced by crystal packing effects (Supporting information 1). In the WT MET•tepotinib structure (PDB-ID: 4R1V), which was solved from cocrystallization in the space group (SG) *P*2_1_, residues of the A-loop and αC-helix are fully defined and involved in crystal packing contacts. In contrast, no crystal packing interactions are found for the A-loops in the Y1235D and F1200I structures, which were obtained by cocrystallization in the same SG *P*2_1_. The Y1234E/Y1235E structure was obtained by soaking into apo crystals and solved in a different SG (F222) but again no crystal packing interactions are found for A-loop residues. It must be noted that a different number of A-loops residues are not defined by electron density in the Y1235D, F1200I, and Y1234E/Y1235E structures indicating protein flexibility (Supporting information 1). The MET•tepotinib crystal structures (WT, Y1235D, F1200I, and Y1234E/Y1235E) comprise αC-helix out/DFG-in conformations based on KinaMetrix and Kincore classifications (33, 34), but the αC-helix is involved in crystal packing contacts in all structures (Supporting information 1). For comparison, A-loop, and αC-helix residues in the double-phosphorylated MET pY1234/pY1235 crystal structures (PDB-ID: 3Q6U and 3Q6W, SG *P*2_1_2_1_2_1_) are not subject of crystal packing contacts and adopt an αC-helix in/DFG-in conformation (Supporting information 1 and 2). Other MET inhibitors crystallized under different buffer conditions in the SG *P*2_1_2_1_2_1_ reveal either αC-helix out/DFG-in (type I inhibitors) or αC-helix in/DFG-out (type II inhibitors) conformations (Supporting information 2). The A-loop is fully defined in most of the MET crystal structures (SG *P*2_1_2_1_2_1_) with type I inhibitors indicating π–stacking interactions to Y1230. In contrast, significant parts of the A-loop are disordered in MET crystal structures (SG *P*2_1_2_1_2_1_) with type II inhibitors and no interactions to Y1230 are visible. We conclude that the observed conformational differences in the MET•tepotinib crystal structures regarding A-loop and αC-helix conformations may not be unduly influenced by crystal packing effects.

### FCCS demonstrated tepotinib binding to full-length MET WT and phosphomimetic mutants from solubilized cellular membranes

**Figure 5:**
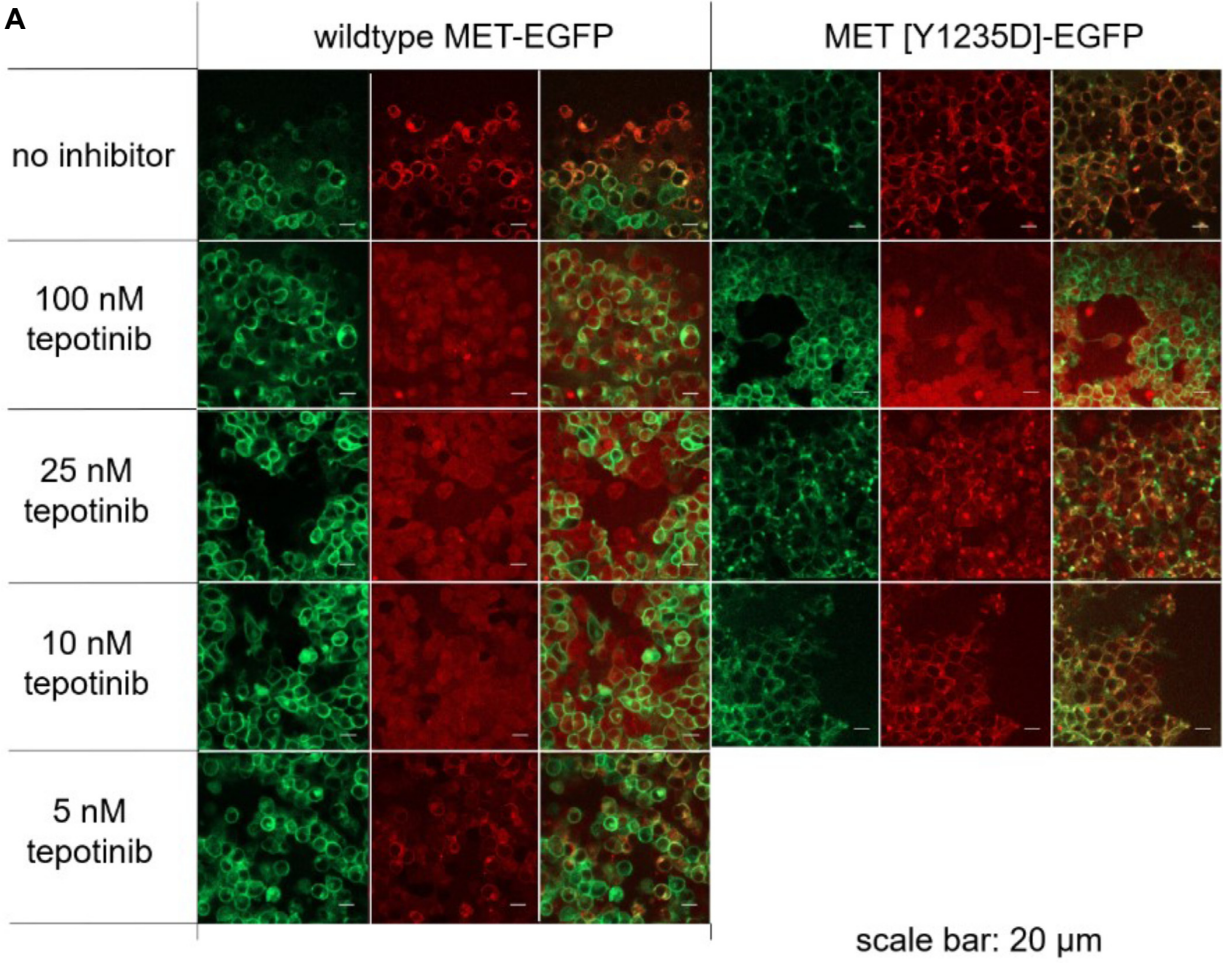

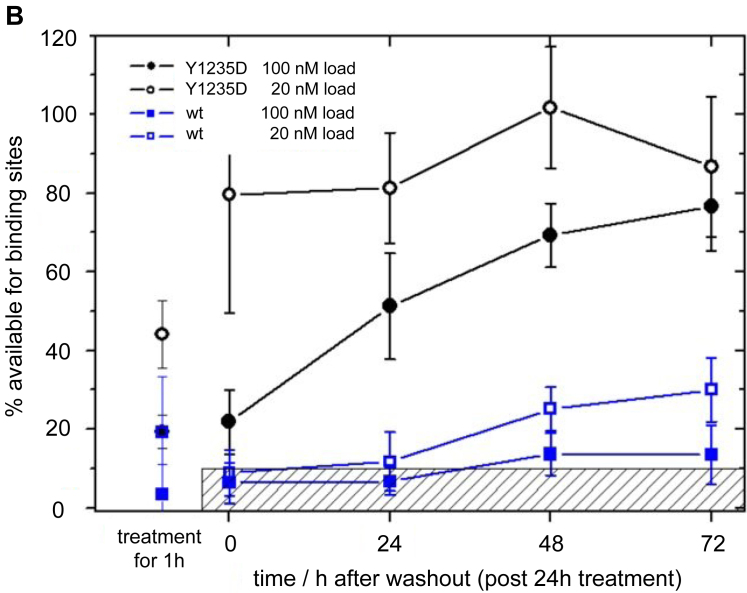
**FCCS demonstrated tepotinib binding to full-length MET WT and phosphomimetic mutants from solubilized cellular membranes.***A*, colocalization of GFP-full length MET receptors (WT and Y1235D mutant) with fluorescent crizotinib tracer in concentration-dependent pretreatment with tepotinib monitored by confocal microscopy of HEK293 cells. GFP fluorescence (*green channel*) is shown in the first and forth column, respectively, indicating the exclusive localization of full-length receptors in the plasma membrane. Localization of the fluorescent tracer (*red channel*) is shown in columns two and five and overlay of both channels in column three and six, respectively. *B*, FCCS target occupancy experiments with WT and Y1235D cells, which were preincubated for 24 h with 20 nM and 100 nM tepotinib and analyzed after wash-out at different time points post treatment. The WT showed a high-target occupancy over 72 h, but the Y1235D mutant was occupied only at higher tepotinib concentrations and showed a time-dependent release of the binding. FCCS, fluorescence cross-correlation spectroscopy; MET, mesenchymal-epithelial transition factor.

We extended our biophysical binding studies to a more physiological context by employing FCCS to investigate target engagement with GFP-labeled KD constructs and full-length receptors expressed in HEK293 cells (35). A fluorescent tracer based on crizotinib (Table S1) (36) was used for affinity determination of MET variants in a FCCS saturation binding assay (Fig. S8*A* and Table S2). Tepotinib showed tight binding affinities to both the WT KD and solubilized full-length receptor, while the phosphomimetic mutants (KD and full-length) indicated reduced affinities (Table 4, Fig. S8*B*). Confocal microscopy revealed the expected exclusive localization of full-length MET in the plasma membrane (Fig. 5*A*). For target engagement, we followed the colocalization of the crizotinib tracer to full-length MET at the plasma membrane. Colocalization was similar for WT and Y1235D expressing cells, indicating that the expressed proteins are binding competent for small molecule inhibitors (Fig. 5*A*). It should be noted that the fluorescent tracer is not cell permeable and can reach the cytoplasmic binding site at the KD only after mild cell permeabilization with 0.05% Triton. In a next step, we confirmed the selective interaction of the full-length MET by competition with tepotinib. Stable cell lines expressing the GFP fusions were preincubated with various tepotinib concentrations and probed for free binding sites with the crizotinib tracer. The colocalization of tracer and receptors was effectively blocked at concentrations of 10 nM tepotinib for the WT but only at 100 nM for the Y1235D mutant (Fig. 5*A*). Our SPR binding kinetic studies have demonstrated prolonged residence time of tepotinib for WT MET in contrast to the Y1234E/Y1235E and Y1235D mutants. Therefore, we explored the kinetic stabilities of tepotinib complexes with native receptors by FCCS under wash-out conditions. Here, we preincubated WT and Y1235D cells with 20 nM and 100 nM tepotinib for 24 h. WT MET samples showed almost complete target occupancy immediately and 24 h after wash-out (Fig. 5*B*). Even 72 h after wash-out only ∼20% more binding sites were available after preincubation with 20 nM tepotinib, while a much smaller increase was observed with 100 nM tepotinib. In contrast, tepotinib complexes with the Y1235D mutant were much less stable. For cells loaded with 20 nM tepotinib a large fraction of ∼80% receptor binding sites was available for tracer binding even directly after the wash-out. After preincubation with 100 nM tepotinib, this fraction was significantly lower (∼20%) and increased gradually to ∼80% after 72 h reflecting the shorter residence time of tepotinib on the Y1235D mutant than the WT.

**Table 4:** Assessment of tepotinib affinity to MET in cell lysates by fluorescence cross correlation spectroscopy

MET protein variants	Tepotinib
	K_i_ (nM)
MET (1028–1390)-EGFP	1.3 ± 0.1
MET (1028–1390) [Y1235D]-EGFP	49 ± 4.9
MET (1028–1390) [Y1234 E/Y1235 E]-EGFP	66
MET (full-length)-EGFP	7.6 ± 1.9
MET (full-length) [Y1235D]-EGFP	75.6 ± 8.1

## Discussion

**Figure 6:**
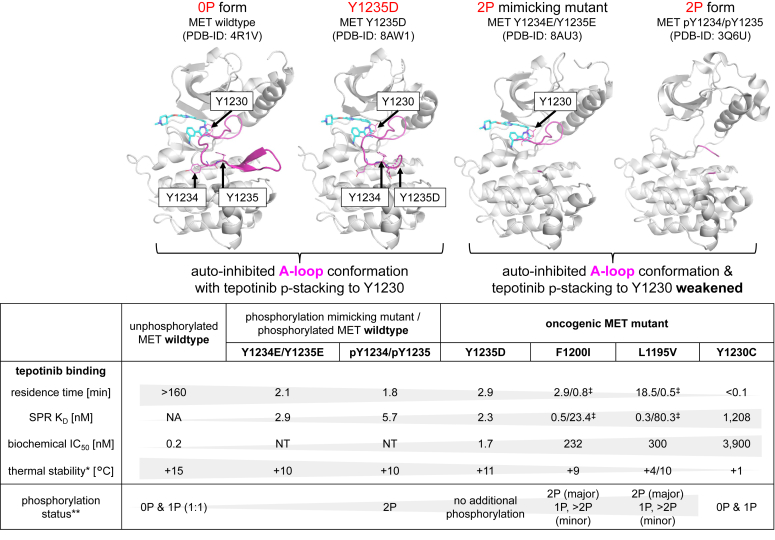
**Biophysical and structural impacts of MET phosphorylation on tepotinib binding.** Crystal structures of MET WT, Y1235D, and Y1234E/Y1235E in complex with tepotinib (*cyan*) showed identical inhibitor positions with π–stacking interactions to Y1230. The low off-rate binding kinetic of tepotinib might be explained by its deeply buried binding position in the unphosphorylated MET WT structure with the A-loop (*magenta*) in an autoinhibited conformation. Our biophysical tepotinib binding studies indicate a link between reduced residence times, lower thermal stabilities, and increased A-loop flexibilities induced either by oncogenic mutations within the A-loop (Y1235D) or in distant positions (L1195V and F1200I), promoting dual Y1234/Y1235 autophosphorylation. The residence time, thermal stability, and binding affinities for tepotinib were further reduced for the oncogenic mutant Y1230C presumably due to enhanced A-loop flexibility including dislodged π–stacking interactions resulting in a more exposed tepotinib binding position. (‡protein mixture contained MET species with high and low SPR K_D_s and residence times for tepotinib binding; ∗thermal stabilities ΔT_m_ (°C) determined by thermal shift assay; ∗∗phosphorylation status on Y1230, Y1234, and Y1235 of recombinant proteins used in tepotinib binding studies analyzed by MS). A-loop, activation loop; MET, mesenchymal-epithelial transition factor; SPR, surface plasmon resonance.

Tepotinib has been introduced as an important new treatment for metastatic NSCLC harboring *MET*ex14 skipping alterations (22, 37). The present study provides a detailed biophysical and structural analysis of the molecular interaction of tepotinib with its target MET. We aim to understand the impacts of kinase activation by A-loop phosphorylation as well as by selected oncogenic mutations on residence time for tepotinib binding. Tepotinib adopts a U-shaped binding conformation in the ATP site of WT MET KD, involving π-stacking contacts to Y1230 of the A-loop, which is unphosphorylated on Y1234 and Y1235 and protrudes into the ATP pocket (Fig. 6). The resulting highly buried binding position of tepotinib in this autoinhibited conformation offers an explanation for the observed slow off-rate binding kinetics corresponding to an estimated residence time of >20 h (38, 39). This binding mode suggests an impeded A-loop phosphorylation, which is supported by the inhibition of MET Y1234 and Y1235 phosphorylation found in tumor biopsy-evaluated patients upon tepotinib treatment (40). Structural comparison with the activated, dual-phosphorylated (pY1235/pY1234) form suggests large conformational shifts of the A-loop, resulting in a more exposed tepotinib binding position (Fig. S9). Indeed, A-loop phosphorylation of both Y1234 and Y1235 resulted in a significantly reduced residence time for tepotinib (Fig. 6). This is further supported by the crystal structure of the phosphomimetic Y1234E/Y1235E mutant, which showed a partly disordered A-loop indicating enhanced protein flexibility but still with identical π-contacts between Y1230 and tepotinib. Accordingly, the Y1235E/Y1234E mutant showed reduced residence time and thermal stability for tepotinib binding in comparison to the unphosphorylated WT. We expected a similar impact on the A-loop conformation for the oncogenic Y1235D mutant based on the H-bond interaction between Y1235 and D1204 as observed in the unphosphorylated WT MET•tepotinib structure (Fig. 1). However, we found an almost identical A-loop conformation with preserved tepotinib-Y1230 π-contacts in the MET Y1235D structure, but surprisingly also positional shifts of Y1234 and Y1235 in comparison to the WT structure (Fig. 6). Again, the partly incomplete A-loop electron density indicates higher protein flexibility in this region of the Y1235D structure in line with the observed lower residence time and melting temperature (T_m_) shift for tepotinib. Our biophysical and structural data are corroborated with FCCS target engagement studies indicating reduced kinetic stability of the MET Y1235D•tepotinib complex, whereas the WT receptor complex was persistent over more than 72 h.

The link between A-loop phosphorylation and reduced residence times or lower T_m_ shifts for tepotinib binding is also evident for the MET F1200I and L1195V mutants. Here we observed an increased presence of higher phosphorylated species (>2P) in the F1200I and L1195V protein samples prior to autophosphorylation and consistent with earlier reports (29). This enhanced phosphorylation status resulted again in decreased residence times and thermal stabilities for tepotinib binding toward F1200I and L1195V (Fig. 6). The reduced biochemical affinities of tepotinib for the F1200I and L1195V mutants are in line with ∼15-fold decreased cellular potencies for tepotinib and other type Ib inhibitors against Ba/F3 cells expressing *MET*ex14 F1200I or L1195V mutants compared with the WT (41). In contrast, MET type II inhibitors are more sensitive to L1195 and F1200 mutations due to direct inhibitor interactions of these residues (41). Based on our tepotinib-bound F1200I crystal structure, we conclude that the F1200I mutation does not directly affect the A-loop conformation and is not involved in tepotinib interactions. The F1200I and L1195V mutations are situated in a hydrophobic cluster at the C-terminal end of the αC-helix (Fig. 4*B*). Structural modification within this hydrophobic cluster by F1200I mutation might explain the shifted αC-helix position and disordered JM-helix observed in the F1200I•tepotinib crystal structure. The F1200I mutation might also indirectly destabilize the A-loop conformation *via* F1134 in connection with the DFG-motif residue F1223 causing an enforced A-loop autophosphorylation. A similar structural impact by F1200I mutation might also be expected for Y1194 and L1195, which are either a known *in vitro* phosphorylation site (Y1194) or a known oncogenic mutation (L1195), respectively (12, 13). The MET L1195V crystal structure in complex with a type Ib inhibitor (PDB ID: 5HOA) adopting a similar binding mode as tepotinib indicated a disordered A-loop as observed in our Y1234E/Y1235E structure (20). Phosphorylation of Y1194 is expected to induce structural rearrangements that would presumably affect the αC-helix position, although experimental evidence is currently missing (12). Taken together we conclude, that the MET F1200I and L1195V mutations promote autophosphorylation resulting in reduced tepotinib affinities and increased dissociation rates due to enhanced A-loop flexibility. This is supported by our SPR data on F1200I and L1195V protein samples with inhomogeneous phosphorylation, which showed both high and low affinity components.

We observed even larger potency drops toward the low micromolar range for tepotinib binding to the Y1230C mutant, which also showed the lowest residence time and thermal stabilization among the protein variants in our study. The Y1230C mutant indicated a mixture of low or highly phosphorylated protein species prior or after ATP incubation similar to that seen for the F1200I mutant. Thus, we attribute the diminished tepotinib binding affinity for the Y1230C mutant mainly to the loss of critical π-stacking contacts between the pyridazinone ring and Y1230 present in the WT, Y1235D, and unphosphorylated F1200I structures. This is supported by significantly reduced cellular potencies of other type Ib inhibitors adopting identical π–stacking interactions to Y1230 as tepotinib against different Y1230 mutants (41). Similar reductions in cellular potencies of type Ib inhibitors were observed for MET mutants in the D1228 position, which is involved in a critical H-bond stabilizing the autoinhibited A-loop conformation (19, 41). In contrast, MET type II inhibitors are almost insensitive to Y1230 and some D1228 mutations because they do not directly interact with these residues (19, 41). Replacement of Y1230 by a cysteine would not only dislodge these π–stacking interactions but might also destabilize the A-loop conformation by impairing H-bond contacts between Y1230, A1226, and K1110 (Fig. S10). Replacement of the pyridazinone ring with an optimized moiety for cysteine interaction might stabilize the A-loop in the C1230 position as observed for the MET Y1230H crystal structure (PDB ID: 5HLW) with a type Ib inhibitor (19).

In summary, we solved the first crystal structures of different MET mutants (Y1235D, Y1234E/1235E, F1200I) with tepotinib and observed identical inhibitor binding positions with critical π–stacking interactions to Y1230 as in the WT structure. We conclude that the low off-rate binding kinetic of tepotinib results from its deeply buried binding position in the unphosphorylated MET WT structure comprising an autoinhibited A-loop conformation. Our biophysical tepotinib binding studies indicate a link between reduced residence times and increased A-loop flexibility induced either by an oncogenic mutation within the A-loop (Y1235D) or by promoting dual Y1234/Y1235 autophosphorylation as observed for oncogenic mutations outside the A-loop (L1195V and F1200I). The residence time and binding affinities for tepotinib were even further reduced for the oncogenic mutant Y1230C presumably due to enhanced A-loop flexibility including dislodged π–stacking interactions with tepotinib. We expect similar reductions of residence time also for other MET type Ib inhibitors with binding modes like tepotinib due to enhanced A-loop flexibility or by Y1230 mutation affecting inhibitor π–stacking interactions. Further biophysical studies on residence time are needed to complement our understanding on other MET inhibitor types and oncogenic mutants in comparison with tepotinib.

## Experimental procedures

### Materials

Recombinant MET KD protein (1051–1349, WT, and mutants) used for SPR, thermal shift assay, and cocrystallization experiments was expressed from insect cells as a GST-fusion protein using the Bac-to-Bac-system. Expression was done in 5 L scale in Sf9 cells using single-use bioreactors. Purification was performed by Affinity capture on GSH-Sepharose, followed by cleavage of the GST-tag, a second affinity chromatography step to remove the cleaved tag, and a final ion exchange chromatography on Resource Q (Cytiva). For crystallization, the protein was concentrated in a final buffer containing 25 mM MES, pH = 6.5, 150 mM NaCl, 2 mM DTT to app. 8 mg/ml. For autophosphorylation of WT MET KD the protein was incubated at a concentration of 75 μM in 30 mM Hepes pH 7.5, 150 mM NaCl, 1 mM tris-(2-carboxyethyl)phosphine, 1 mM Na_3_VO_4_ with 2 mM ATP/Mg for 5 min at room temperature (RT). The reaction was stopped by addition of EDTA to a final concentration of 5 mM.

### Native iso-electric focusing gel electrophoresis

Changes of the isoelectric point of MET KD protein due to autophosphorylation was assessed by native IEF electrophoresis using the Novex gel electrophoresis system (Invitrogen/Thermo Fisher Scientific) with pH 3 to 10 IEF Protein Gels, 1 mm, and premixed buffer systems according to vendor instructions. As reference the IEF Marker3-10 from Serva was used. The electrophoreses was executed with increasing voltage steps (60 min at U = 100 V; 60 min at U = 200 V; 30 min at U = 500 V). Gels were fixed in 20% trichloro acetic acid for 30 min and stained with Coomassie Instant blue according to manufacturer instructions.

### Bioanalytical HPLC

Changes of the surface charge properties of MET KD protein due to autophosphorylation was assessed by weak cation exchange chromatography using a biocompatible Bioanalytical HPLC system (JASCO GmbH Groß-Umstadt) equipped with a DIONEX ProPac WCX-10 (4 × 250 mm) column. Samples of recombinant proteins or from autophosphorylation reactions were loaded on the column equilibrated in 20 mM sodium phosphate (pH 6) and 200 μM Na_3_VO_4_ as mobile phase (flow 1 ml/min) and eluted in a linear gradient with NaCl concentration increasing to a maximum of 500 mM within 20 min, followed by an isocratic elution in buffer with 500 mM NaCl for another 8 min. The elution of proteins was monitored *via* the change in UV absorbance (λ = 280 nm, 257 nm, and 211 nm) as well as *via* tryptophan fluorescence (excitation wavelength λ = 280 nm; recorded emission wavelength λ = 350 nm).

### Differential scanning fluorimetry

The protein T_m_ determination was performed by monitoring protein unfolding with the fluoroprobe SYPRO Orange dye (Molecular Probes), which although completely quenched in aqueous environment, emits fluorescence upon binding to protein hydrophobic patches. Such increase in fluorescence can be measured as a function of temperature. The DSF assay was performed on an iCycle iQ5 Real-Time PCR Detection System (Bio-Rad), equipped with a charge-coupled device camera and a Cy3 filter with excitation and emission wavelengths of 490 and 575 nm, respectively. This equipment can simultaneously detect the fluorescence changes in 96-well plates (low-profile plate, Bio-Rad), and thus can be used for parallel thermal stability assays. The 96-well plates were sealed with optical quality sealing tape (Bio-Rad) and centrifuged at 2500*g* for 2 min immediately before the assay to remove possible air bubbles. The plates were subsequently heated from 20 °C to 90 °C with stepwise increments of 1 °C with a 60 s equilibration time, followed by the fluorescence read out. In a typical assay with a total volume of 20 μl, a protein concentration of 0.125 mg/ml and a dye concentration of 5-fold were used to guarantee the best signal to noise ratio. The assay was prepared by adding 5 μl of protein to 15 μl of dye buffer solution, all prepared in the protein purification buffer. The tepotinib treated sample was previously prepared by incubating MET with ligand for 3 h at 4 °C (final concentration is 20 μM and 5% dimethyl sulfoxide (DMSO)). Fluorescence intensities *versus* temperature are used to calculate the protein T_m_ by determining the first derivative (d(Rfu)/dT) to extract the exact transition inflection point.

### Surface plasmon resonance

The kinetic and affinity parameters of the interaction between MET WT and mutants with tepotinib was assessed by SPR. MET WT and mutants were immobilized onto a CM5 (Series S) sensor chip *via* the standard amine coupling procedure, at 25 °C. Prior to immobilization, the carboxymethylated surface of the chip was activated with 400 mM 1-ethyl-3-(3-dimethylaminopropyl)-carbodiimide and 100 mM N-hydroxysuccinimide for 10 min. MET WT and mutants were diluted to 5 μg/ml in 10 mM Na-phosphate at pH 7 and immobilized on the activated surface chip in order to reach 700 to 1200 response units. The remaining activated carboxymethylated groups were blocked with a 7 min injection of 1 M ethanolamine pH 8.5 after which the surface chip was washed with 0.5% (w/v) SDS and 50 mM glycine. HBS-N (0.1 M Hepes, 1.5 M NaCl) was used as the background buffer during immobilization. All compounds were prediluted in DMSO, diluted 1:50 in running buffer (20 mM Hepes pH 7.4, 150 mM NaCl, 1 mM DTT, 2 mM MgCl_2_, 0.1 mM EGTA, 0.05% Tween 20) and injected at ten different concentrations using 2-fold dilution series, from 0.5 μM to 0.001 μM. A DMSO solvent correction (1%–3%) was performed to account for variations in bulk signal and to achieve high-quality data. Interaction analysis cycles consisted of a 150 s sample injection (30 μl/min; association phase), followed by 300 s of buffer flow (dissociation phase). All sensorgrams were processed by first subtracting the binding response recorded from the control surface (reference spot), followed by subtracting of the buffer blank injection from the reaction spot. All datasets were fit to a simple 1:1 Langmuir interaction model to determine the kinetic rate constants. Experiments were performed on a Biacore 8k+ (Cytiva) at 25 °C, and the interactions were evaluated using the provided Biacore Insight evaluation software (https://www.cytivalifesciences.com/en/us/support/software/biacore-downloads/biacore-insight-evaluation-software).

### Fluorescence cross correlation spectroscopy

#### Fluorescent labeling of crizotinib with PEG4-DY647

Crizotinib (36) was dissolved and mixed in DMSO and labeled *via* the reactive amino group to PEG4-DY-647 (Dyomics). The mixture was incubated in the dark at RT for 2 h. The conjugate was purified by reversed-phase HPLC (HP-1100, Agilent) using a linear gradient of water and acetonitrile. The resulting crizotinib based probe was lyophilized, dissolved in DMSO, and stored at −20 °C prior to the use in a binding assay or in imaging experiments.

#### Cell culture conditions

HEK293 cells were cultured in Dulbecco's Modified Eagle Medium with high glucose (Cat. No 41966-029; Gibco) supplemented with 10% (v/v) fetal calf serum (Cat. No. S0115; Biochrom). Cells were grown at 5% CO_2_ and 37 °C in a humidified incubator.

#### Expression of MET-WT-eGFP and MET-Y1235D-eGFP

MET full-length WT coding gene was cloned as C-terminal GFP fusion in a mammalian expression vector pCGTO (a derivative of pcDNA3.1, Invitrogen, Cat. No. V860-20) under the control of the tetracycline promoter and expressed in HEK293 cells. Mutation Y1235D was generated by using Q5 Site-Directed Mutagenesis Kit (NEB, Cat. No. E0554S). Sequences were confirmed by Sanger sequencing. Correct expression of MET proteins was confirmed by fluorescence microscopy and Western blotting. Typically, HEK293 cells (5 × 10^6^) were transfected with plasmids encoding GFP-tagged protein using the NanoFectin transfection kit (PAA, Cat. No. Q051-005). Transfected cells were investigated microscopically or harvested 24 h after the transfection. During the harvest, the cells were washed twice with PBS, pelleted for 5 min at 1100*g*, and then frozen at −80 °C.

#### Membrane preparation and receptor solubilization

Membrane preparation from HEK293 cells expressing MET-eGFP were performed as per Antoine *et al*. (42) Membrane preparation was then diluted with Tris-buffered saline (50 mM Tris/HCl pH 7.4, 150 mM NaCl) supplemented with protease inhibitor cocktail (Roche #04906845001) (henceforth termed Tris-buffered saline/PI) to a concentration of 1 mg/ml (total membrane protein), and a mixture of detergents n-dodecyl b-D-maltoside/CHAPS/cholesteryl hemi succinate was added at their final concentrations of 0.25%, 0.5%, or 0.1% (w/v), respectively. The membrane preparation was solubilized for 1 h at 4 °C with end-over-end rotation. Insoluble material was pelleted by centrifugation for 1 h at 100,000*g* at 4 °C. Supernatant containing solubilized MET-eGFP was used in FCCS experiments, which were carried out at RT.

### FCCS measurements

All FCCS measurements were performed as described previously (42, 43). Briefly, saturation binding assays were first carried out to determine the equilibrium dissociation constants of MET-WT-eGFP and MET-Y1235D-eGFP and crizotinib–PEG4-DY647 interaction. Then, Ki-values were determined from inhibition binding-data by using a previously described four-parameter logistic function (42). All the experiments were carried out after incubating the samples for 1 h at RT while shaking. Data acquisition for samples in equilibrium typically required 20 to 60 s per sample.

#### Live-cell imaging/confocal microscopy

Confocal laser scanning microscopy was conducted on an LSM510 confocal microscope as previously described (42, 43). Cells were preincubated with different concentrations of tepotinib for 1 h. Cells were then washed once with PBS, then 50 nM crizotinib-PEG4-DY647 were added to the cells in a solution of PBS with or without 0.05% Triton to allow the permeabilization of the cell membrane. Images were acquired after 10 min incubation at RT.

#### Target occupancy measurement

Plates of HEK293 cells expressing MET-WT-eGFP and MET-Y1235D-eGFP were cultured until ∼80% confluency. Then cells were treated with 20 nM or 100 nM concentration of tepotinib for either 1 or 24 h. Samples treated for 1 h were directly collected and frozen. Samples treated for 24 h were washed with PBS and then one plate was collected for time 0 sample. The other plates were used to seed cells in order to get ∼80% confluent plates after 24-, 48-, and 72-h incubation, respectively. Samples were frozen at −80 °C, and lysis and membrane solubilization was performed as previously reported. After solubilization samples were all diluted to 20 nM in Tris-buffered saline-PIC (1× DC) and tested at FCCS with 50 nM crizotinib-PEG4-DY647 with or without 100 μM tepotinib. Incubation for 1 h at RT, 12 measurement ∗5 s.

#### NanoDSF

NanoDSF measurements were performed with a Prometheus NT.Plex from NanoTemper. For all nanoDSF experiments, the MET KD before and after autophosphorylation and the respective compounds were formulated in 50 mM NaPi, 50 mM NaCl, 1 mM tris-(2-carboxyethyl)phosphine, and 0.01% Tween 20 pH 7. The final assay concentration of the proteins was 0.1 mg/ml and for the compounds 50 μM in buffer. All buffers were adjusted to a final concentration of 1% (v/v) DMSO. Samples were loaded into high-sensitivity capillary chips and the thermal unfolding was recorded in a temperature interval from 20 °C to 95 °C and a ramp of 1 °C/min. Fluorescence changes were monitored at 330 nm or 350 nm and unfolding transitions were fit to a two-state model using the PR.Stability Analysis software (NanoTemper, www.nanotempertech.com) to determine the T_m_. Changes in T_m_ in presence of compounds are calculated by subtracting the T_m_ of the DMSO control.

### Mass spectrometry

#### In-gel digestion

The selected, excised gel bands were prepared using a published protocol (44). Reduction, alkylation, and tryptic digestion were performed as described. At the end, the digested samples were completely dried and pending further processing stored at −20 °C.

#### Phosphopeptide enrichment

With the aim to detect possible phosphopeptides, a less complex sample is required by enrichment of phosphopeptides and removal of nonphosphopeptides. Therefore, a part of each digested sample was enriched with the ProteoExtract Phosphopeptide Enrichment TiO_2_ Kit (Calbiochem). The samples were processed as described in the manufacturer’s guide.

#### High-performance liquid chromatography-MS

The tryptic digests were separated and analyzed by Nano-LC-MSe using a nanoAcquity ultra performance liquid chromatography coupled to a Synapt G1 HDMS (Waters) operated in MSe mode. Formic acid (0.1%) in water was used as solvent A and 0.1% formic acid in acetonitrile as solvent B. Tryptic peptides were injected and trapped for 4 min on a Symmetry C18 precolumn (5 μm, 180 μm × 20 mm, Waters) with a flow rate of 10 μl/min. Separation was performed using an ultra performance liquid chromatography 1.7 μm BEH130 column (C18, 75 μm × 100 mm, Waters) with a flow rate of 450 nl/min, starting with 3% B, followed by a linear gradient to 45% B for 70 min. The following washing step was done with 95% B for 5 min and column regeneration with 3% B for 15 min. The HPLC outlet was coupled to a nano source with lock spray inlet. The data acquisition was done for 90 min in positive-, V-, and MSe fragmentation mode. This mode switches between a normal MS acquisition and a high energy fragmentation mode every second. The MS survey range was recorded between 50 and 1600 *m/z* with 1 s scan time. Low-energy setting was 5V, the ramp energy was set from 15 to 40 V. GluFibrinopeptide B human (F3261, Sigma) was used for prior- and lock spray-calibration (1 s scan time every 30 s).

#### Data analysis

Data deconvolution and processing with done with BiopharmaLynx (Waters) software (https://www.waters.com) including the MET amino acid sequence. The carbamidomethyl modification (C) was set as fixed modification. Deamidation (NQ), oxidation (M), and phosphorylation (STY) were used as variable modification. For the relative quantification, the absolute intensity of a peptide of interest was calculated in relation to the average value of the absolute intensities of the five highest MET peptides of the same LC-MS experiment.

### Protein crystallization

For cocrystallization of tepotinib in complex with MET Y1235D or MET F1200I, protein solution (9 mg/ml with 2 mM tepotinib) was mixed in a 1:1 ratio with reservoir solution (8%–18% (w/v) PEG4000, 11%–17% (v/v) isopropanol) at 293 K according to published protocols (45). The crystal structure of MET Y1234E/Y1235E in complex with tepotinib was obtained by soaking apo crystals for 2 h with 0.5 mM tepotinib. MET Y1234E/Y1235E apo crystals were obtained by mixing protein (at 5 mg/ml in 25 mM PIPES pH = 6.5, 150 mM NaCl, 5% glycerol, 1 mM tris-(2-carboxyethyl)phosphine) in a 1:1 ratio with reservoir solution (0.10 M MES pH = 6.50, 3.4 M sodium formiate) at 293 K. Before flash cooling in liquid nitrogen, crystals were transferred to the cryoprotectant (12% PEG4000, 15% isopropanol, 150 mM NaCl, 17% ethylene glycol, and 50 mM MES pH 6.4). Diffraction data were collected at 100 K at the Swiss Light Source (Villingen). Data were processed using the programs XDS and XSCALE (MET Y1235D and MET F1200I) or AutoProc (MET Y1234E/Y1235E), and structures were solved with phaser (46, 47, 48). Several rounds of manual model building in COOT and bulk solvent correction, positional, B-factor, and translation/libration/screw refinement using REFMAC yielded the final models (49, 50). Data collection and model statistics are shown in Table 3.

## Data availability

The structures reported in this paper have been deposited in the Protein Data Bank with the accession codes of 8AU3 for MET Y1234E/Y1235E•tepotinib, 8AW1 for MET Y1235D•tepotinib, and 8AU5 for MET F1200I•tepotinib. All other data are available from the authors upon reasonable request. Any requests for data by qualified scientific and medical researchers for legitimate research purposes will be subject to the healthcare business of Merck KGaA, Darmstadt, Germany, Data Sharing Policy. All requests should be submitted in writing to the healthcare business of Merck KGaA, Darmstadt, Germany, data sharing portal (https://www.emdgroup.com/en/research/our-approach-to-research-and-development/healthcare/clinical-trials/commitment-responsible-data-sharing.html). The atomic coordinates and structure factors have been deposited in the Protein Data Bank (www.pdb.org) with PDB ID codes 8AU3, 8AW1, and 8AU5.

## Supporting information

This article contains supporting information.

## Conflict of interest

The authors declare that they have no conflicts of interest with the contents of the article.
