# Supplementary material for: Biophysical and structural characterization of the impacts of MET phosphorylation on tepotinib binding
Source: J Biol Chem. 2023 Oct 6;299(11):105328. doi: 10.1016/j.jbc.2023.105328 (PMC10654029; doi:10.1016/j.jbc.2023.105328)
Supplement: Supporting Figures S1–S10 and Tables S1 and S2 [file mmc1.docx]

Supporting information for

*Biophysical and structural characterization of the impacts of MET phosphorylation on tepotinib binding*

Ulrich Grädler^1*^, Daniel Schwarz^1^, Ansgar Wegener^1^, Thomas Eichhorn^2^, Tiago M. Bandeiras^3^, Micael C. Freitas^3^, Alfred Lammens^4^, Oleg Ganichkin^4^, Martin Augustin^4^, Stefano Minguzzi^5^, Frank Becker^5^, Jörg Bomke^1^

^1^The healthcare business of Merck KGaA, Frankfurter Str. 250, 64293 Darmstadt, Germany.

^2^Merck KGaA, Frankfurter Str. 250, D-64293 Darmstadt, Germany

^3^iBET, Instituto de Biologia Experimental e Tecnológica, Apartado 12, 2781-901 Oeiras, Portugal.

^4^Proteros biostructures GmbH, Bunsenstraße 7A, 82152 Planegg, Germany.

^5^Intana Bioscience GmbH, Lochhamer Str. 29a, 82152 Planegg, Germany.

*Corresponding author Tel.: +49-6151-725975, Fax: +49-6151-72915975, E-mail: ulrich.graedler@emdserono.com


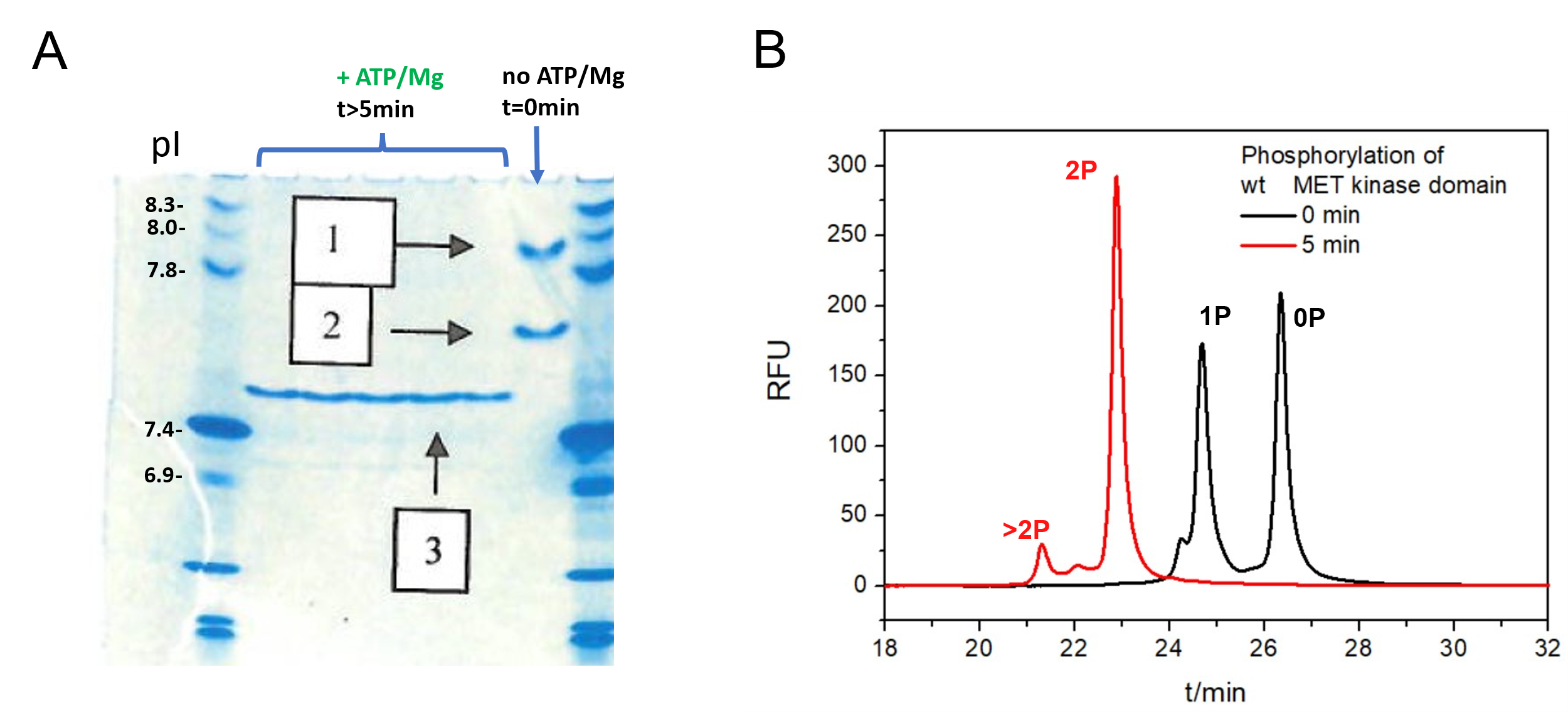

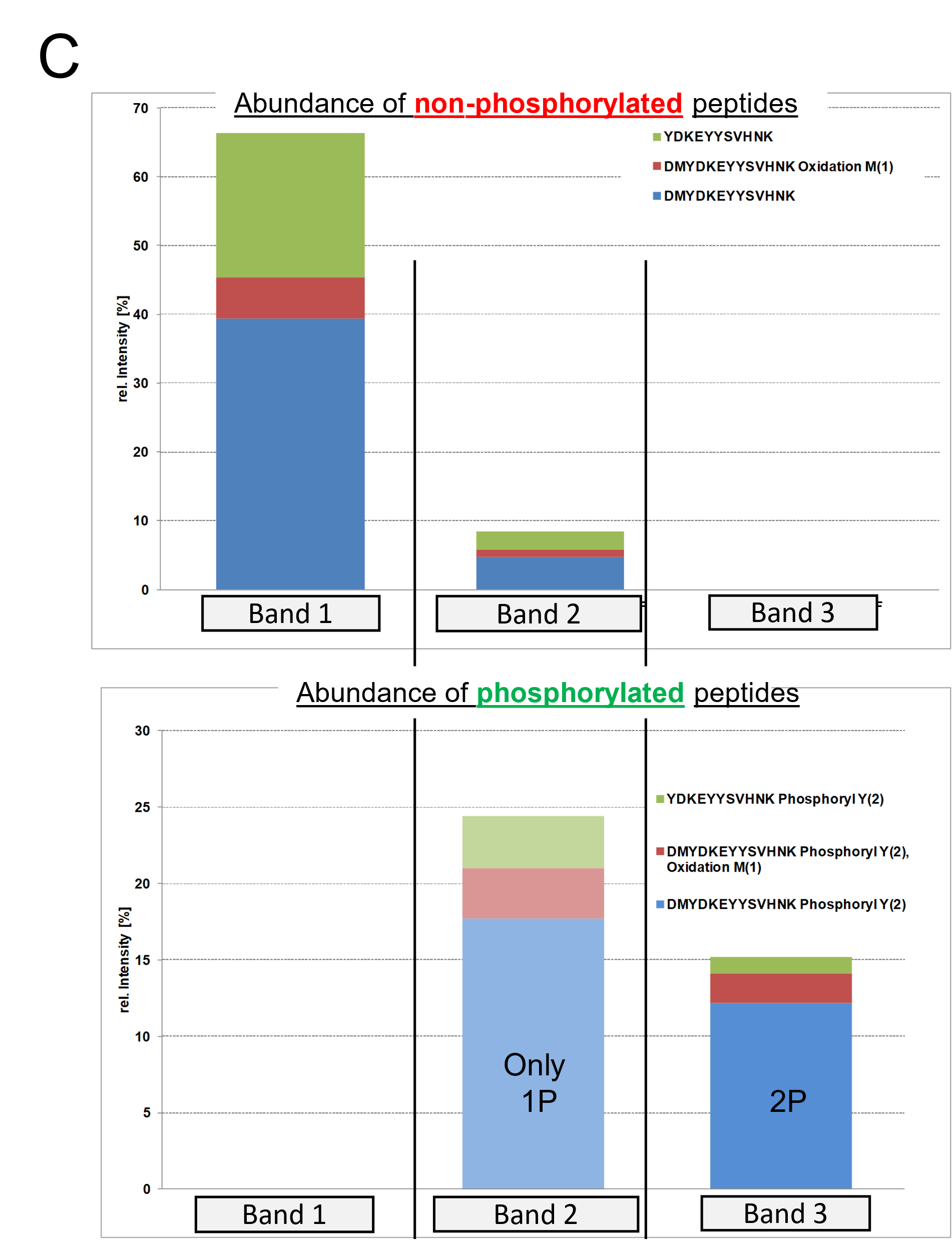


**Figure S1**. (A) Native IEF gel (marker protein on left lane with indicated pI values) of purified recombinant wildtype MET kinase domain protein identified two major bands 1 and 2 prior to ATP/Mg incubation (t=0) and band 3 after >5 min incubation with ATP/Mg^2+^ (B) Weak cation exchange chromatography with bioanalytical HPLC. Overlay of chromatograms of purified recombinant wildtype MET kinase domain protein before (black) and after 5 min incubation with ATP/Mg^2+^ domain protein (red). Phosphorylation status of proteins derived in MS analysis is indicated by labels next to protein bands and elution peaks. (C) The bands were analyzed by mass spectrometric peptide mapping and revealed only non-phosphorylated protein in band 1, mostly mono-phosphorylated protein (pY1234) in band 2 and mainly dual-phosphorylated protein (pY1234/pY1235) in band 3.


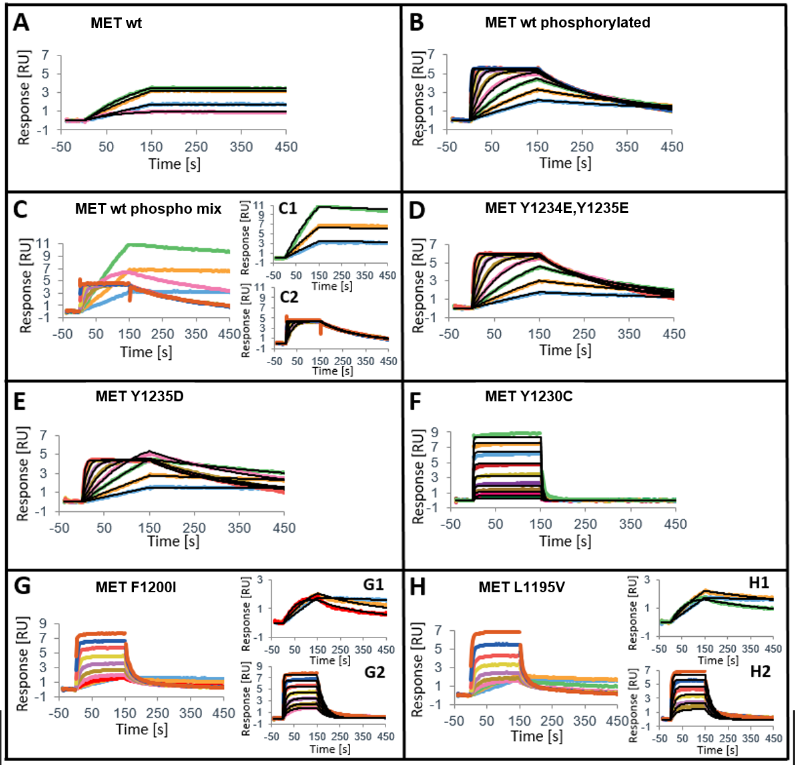


**Figure S2**. Experimental SPR sensorgram overlay plots from an injection series of tepotinib to a MET (1051-1349) wildtype protein surface performed on a Biacore 8k+ instrument. MET protein was immobilized either without ATP incubation (A) or after 5 minutes of ATP incubation (autophosphorylation reaction stopped by EDTA) (B). Additionally, sensorgram overlay plots for MET Y1234E/Y1235E (D), MET Y1235D (E), and Y1230C (F) are displayed. Colored curves represent the recorded data and are overlaid with a 1:1 Langmuir binding model kinetic fit (black). MET wildtype protein comprising of a mix of phosphorylated species (C) was immobilized, as well. Colored curves represent the recorded data and could not be overlaid with a 1:1 Langmuir binding model. Sensorgram overlay plots have been separated into two sub-populations: one representing the high-affinity species (low degree of phosphorylation) (C1) and corresponding low-affinity species (higher degree of phosphorylation) (C2). The overlay plots representing the protein sub-populations have been overlaid with a 1:1 Langmuir binding model (black). Recorded data for MET F1200I (G) and MET L1195V (H) similarly could not be overlaid with a 1:1 Langmuir binding model. Sensorgram overlay plots have been separated into two sub-populations: one representing the high-affinity species (low degree of phosphorylation) (G1 and H1) and the corresponding low-affinity species (higher degree of phosphorylation) (G2 and H2). The overlay plots representing the protein sub-populations have been overlaid with a 1:1 Langmuir binding model (black). The top concentration is 500 nM and injections are related by 2-fold dilutions in 10 steps. The K_D_ is taken as the ratio of the dissociation rate constant (k_d_) divided by the association rate constant (k_a_).


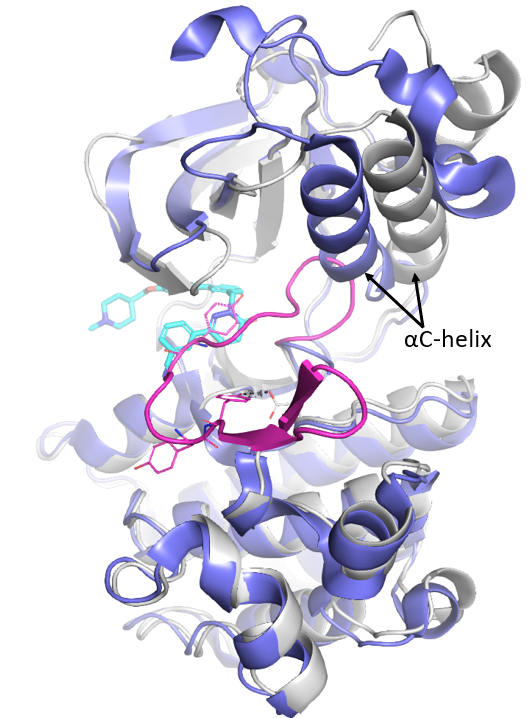


**Figure S3**. Overlay of MET crystal structures of the unphosphorylated wildtype complex with tepotinib (grey, PDB-ID: 4R1V) and the dual-phosphorylated apo form (blue: PDB-ID: 3Q6U) revealed different αC-helix positions indicating an inactive KD conformation present in the tepotinib complex.

**
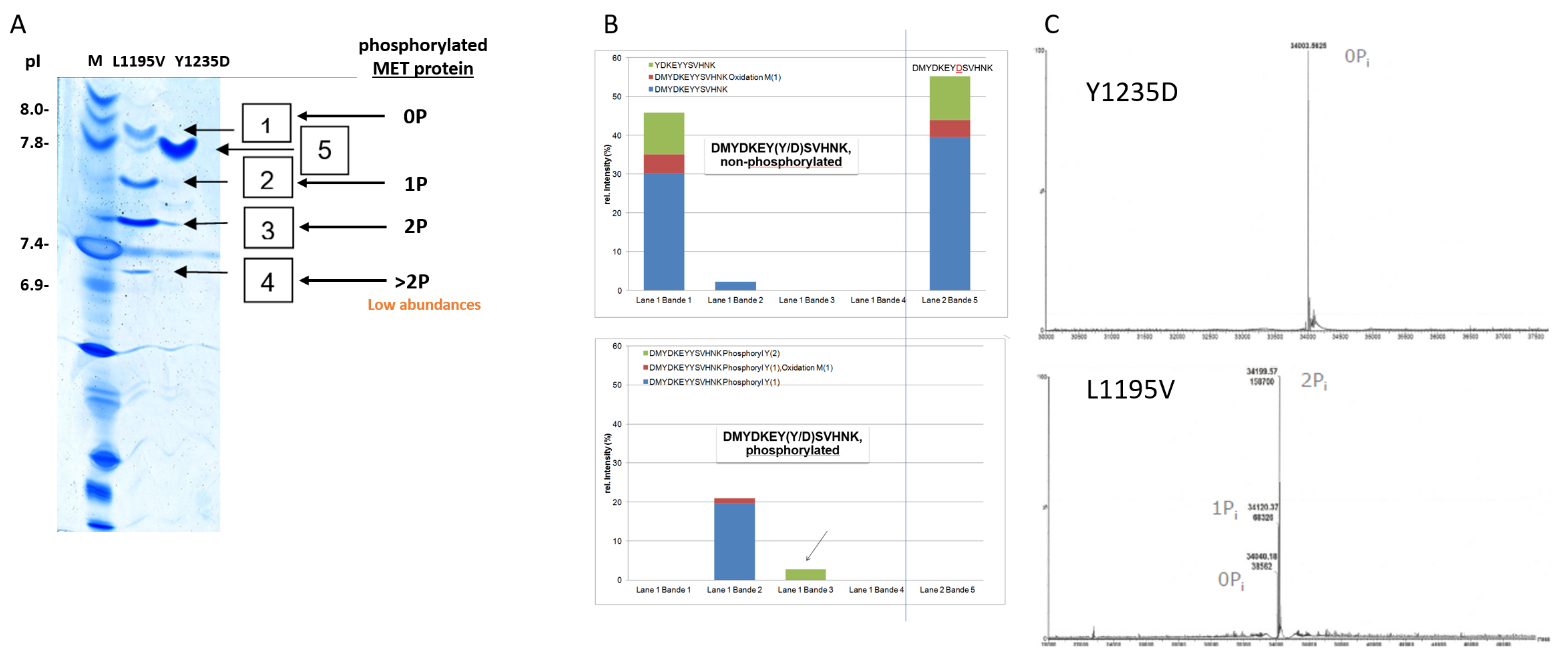
**

**Figure S4**. (A) Native IEF gel of MET (1051-1349) L1195V and Y1235D mutants. (B) MS analysis of phosphorylated tyrosine residues (Y1230, Y1234 or Y1235) present in peptide DM**Y^1230^**DKE**Y^1234^Y^1235^**SVHNK obtained from tryptic digestion of band 5 from SDS gel with Y1235D protein. (C) ESI-MS data for L1195V and Y1235D mutants.


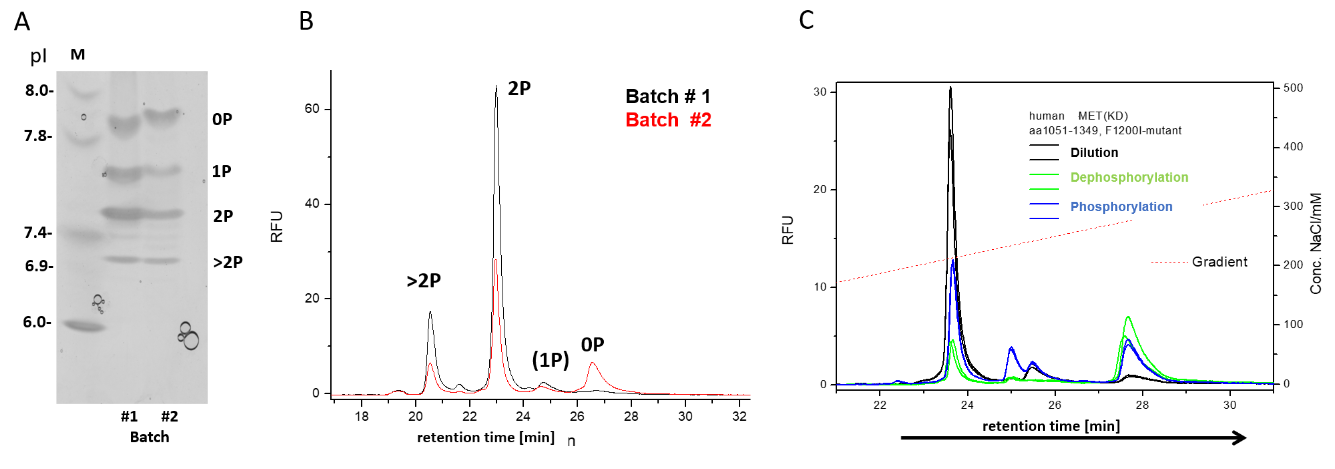


**Figure S5**. (A) Native IEF gel of two different recombinant MET F1200I protein batches showed four main bands at pI values of ~7.9, ~7.6, ~7.5 and ~7.1, which correspond to HPLC peaks (B) at retention times of ~20.5, ~23, ~24.5 and ~26.5 minutes (overlay of two HPLC runs for the two different batches colored in black and red). (C) *In vitro* phosphorylation and dephosphorylation experiments with F1200I protein allowed to assign the HPLC peak at highest retention time (~27.5 min) as 0P form and the two major HPLC peaks at lowest retention times (~23.5 min and ~25 min) as higher phosphorylated species (2P and >2P).


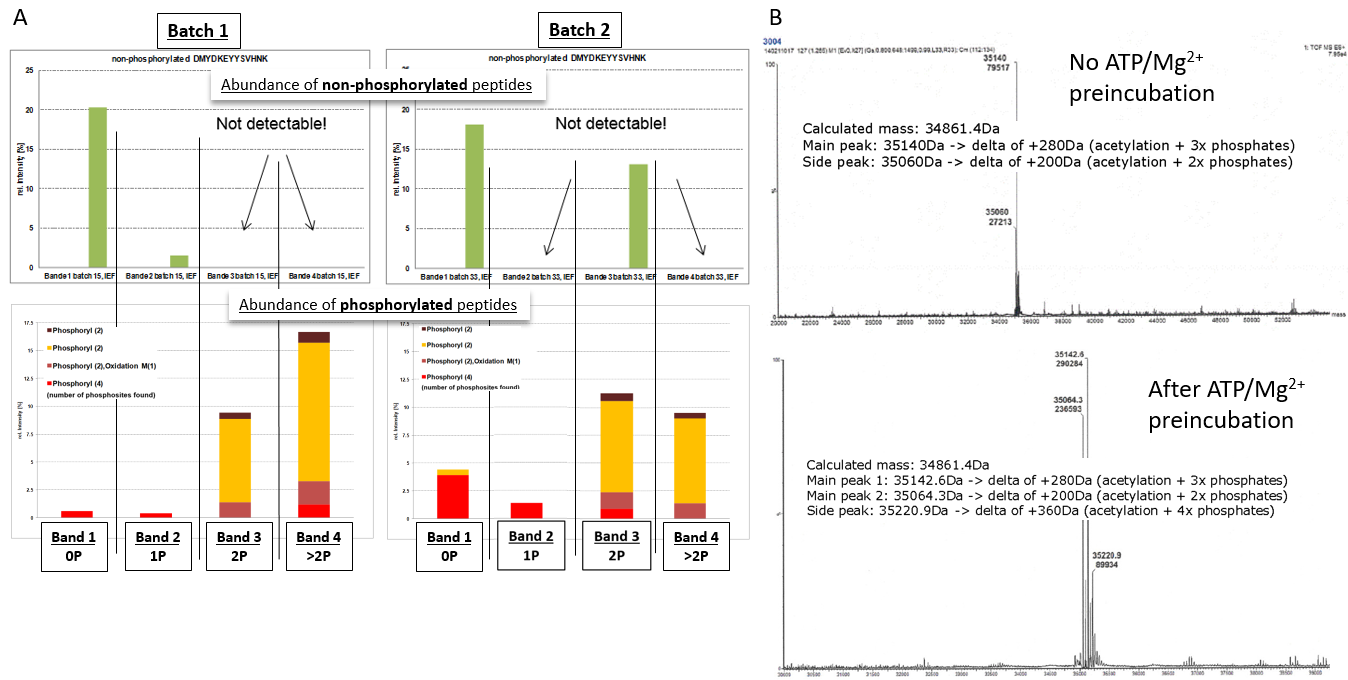


**Figure S6**. (A) MS analysis of the four major MET F1200I IEF bands revealed the following abundances non-phosphorylated and phosphorylated tyrosine residues (Y1230, Y1234 or Y1235) present in peptide DM**Y^1230^**DKE**Y^1234^Y^1235^**SVHNK obtained from tryptic digestion: 0P form at pI~7.9, 1P form at pI~7.6, 2P form at pI~7.5 and >2P forms at pI~7.1. (B) ESI-MS analysis of untreated F1200I protein showed the presence of the 2P and 3P forms, while the protein after in vitro phosphorylation by ATP incubation contained 2P, 3P and 4P forms.


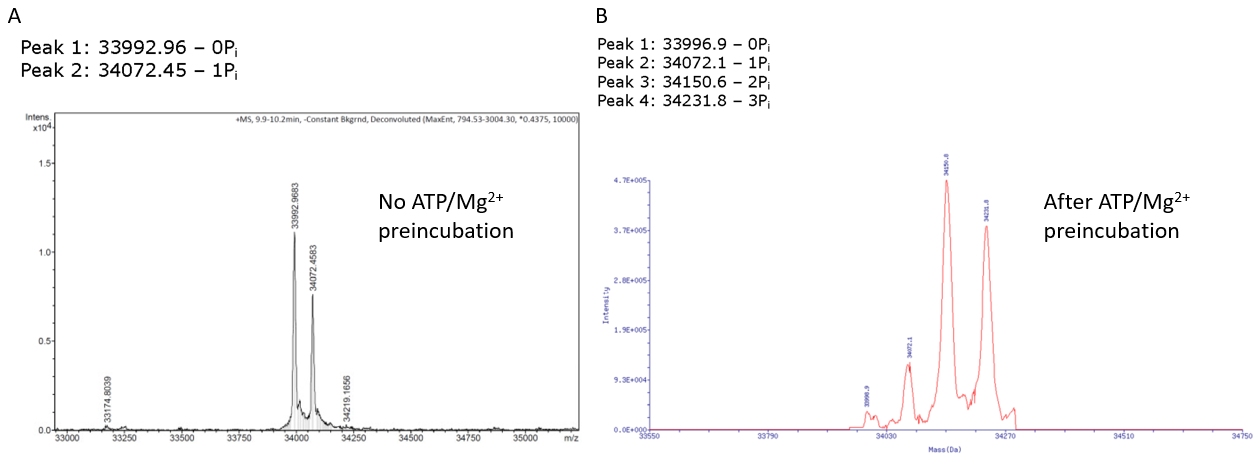


**Figure S7**. (A) ESI-MS data for Y1230C mutant prior to ATP/Mg^2+^ preincubation revealed the presence of 0P and 1P forms. (B) ESI-MS data for Y1230C mutant after ATP/Mg^2+^ preincubation showed the presence of 0P, 1P, 2P and 3P forms.

A
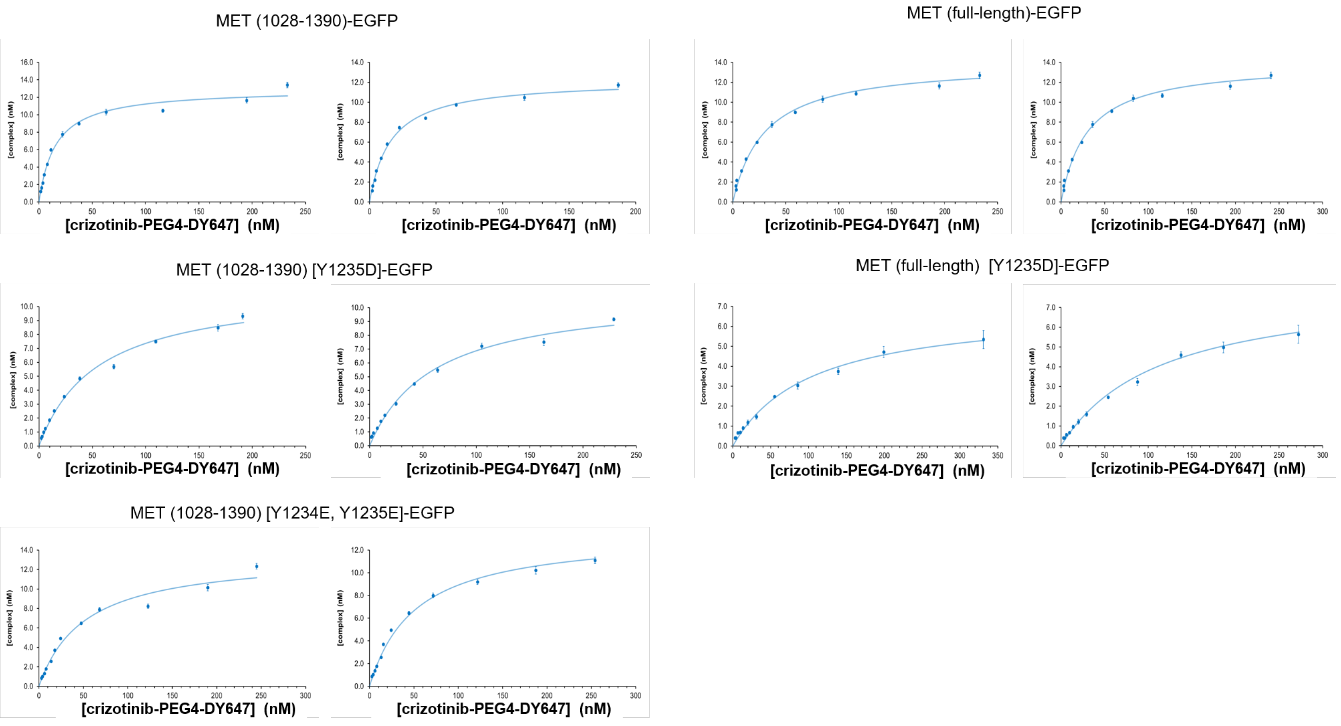


**Figure S8**. (A) Saturation binding for titration of crizotinib-PEG4-DY647 to MET variants.

B
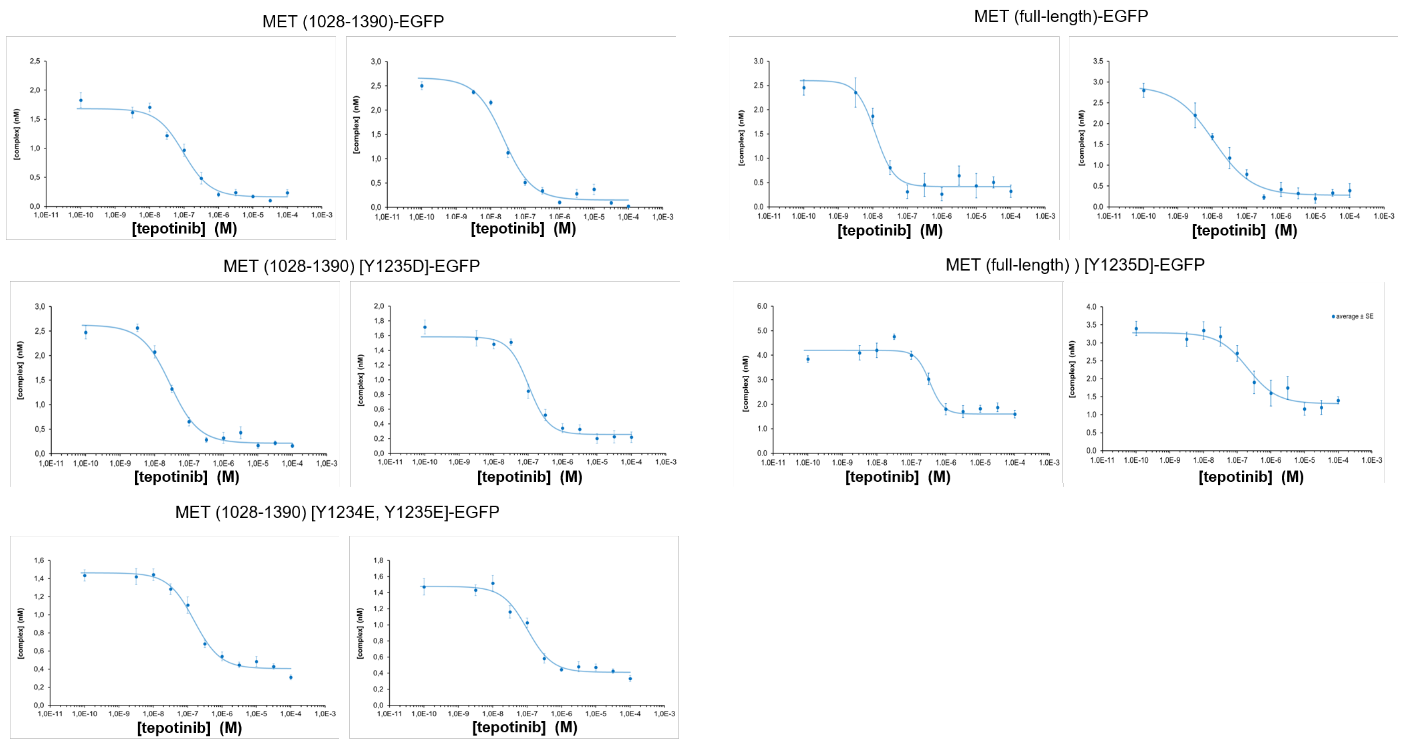


**Figure S8**. (B) Dose response curves for displacement of crizotinib-PEG4-DY647 from MET variants by increasing concentration of tepotinib.


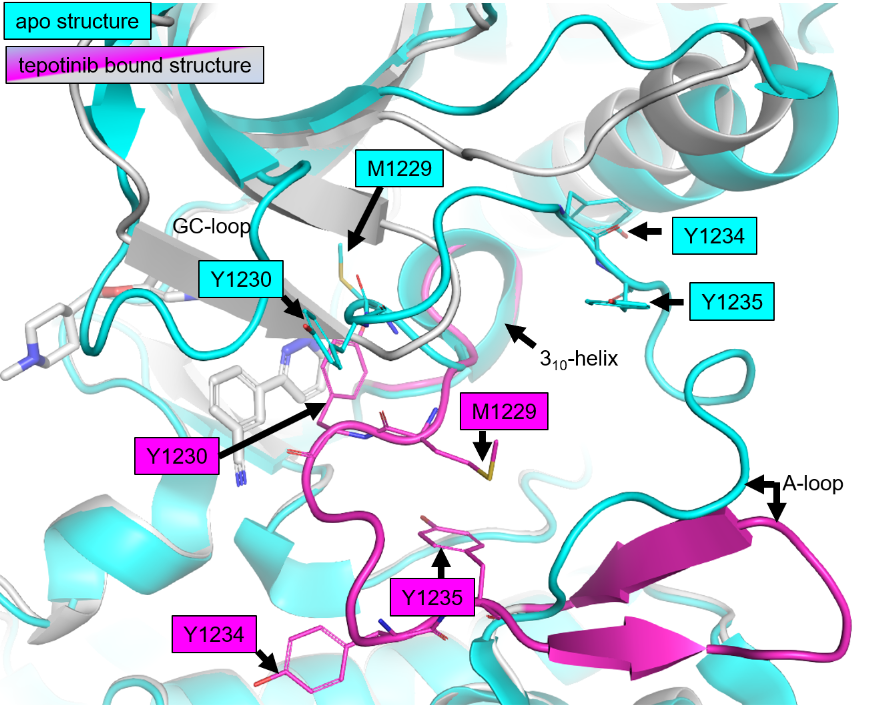


**Figure S9**. Overlay of MET crystal structures of the unphosphorylated wildtype complex with tepotinib (grey, A-loop in magenta, PDB-ID: 4R1V) and unphosphorylated MET apo form (cyan: PDB-ID: 2G15) revealed similar A-loop conformations regarding the short 3_10_ helix (residues G1224 to R1227), but large conformational differences of A-loop residues 1229 to 1245 including M1229, Y1230, Y1234 and Y1235.


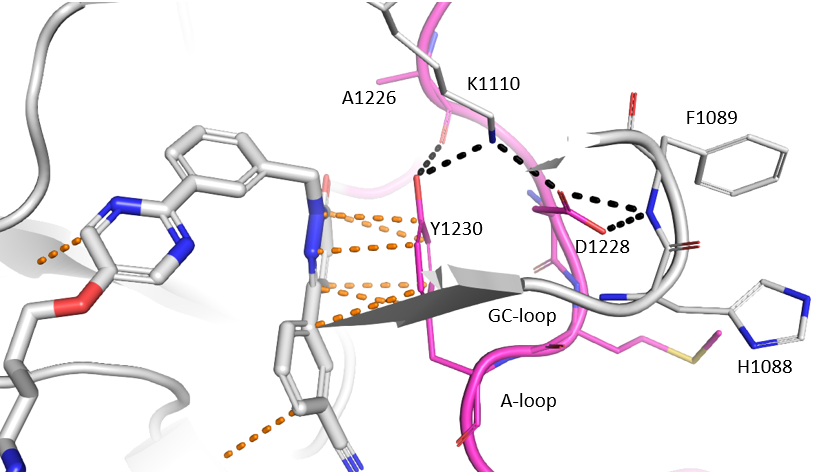


**Figure S10**. H-bond interactions (dashed black lines) observed in the MET wildtype crystal structure with tepotinib (grey, A-loop in magenta, PDB-ID: 4R1V) involving Y1230 (π-stacking contacts shown as dashed orange lines), A1226 and K1110, which is connected via D1228 to the GC-loop (H1088, F1089).

**Table S1**. Chemical structures of compounds used in this study.

| Compound | structure |
| --- | --- |
| tepotinib |  |
| crizotinib |  |

**Table S2:** K_D_-values for crizotinib-PEG4-DY647 binding to MET variants determined in saturation assays (Figure S8A) in cell lysates or with solubilized membranes by fluorescence cross correlation spectroscopy**.**

| MET protein variant | crizotinib-PEG4-DY647 |
| --- | --- |
|  | K_D_ (nM) |
| MET (1028-1390)-EGFP | 15 |
| MET (1028-1390) [Y1235D]-EGFP | 57 |
| MET (1028-1390) [Y1234E, Y1235E]-EGFP | 62 |
| MET (full-length)-EGFP | 30 |
| MET (full-length) [Y1235D]-EGFP | 110 |
