# Supplementary material for: Biophysical and structural characterization of the impacts of MET phosphorylation on tepotinib binding
Source: J Biol Chem. 2023 Oct 6;299(11):105328. doi: 10.1016/j.jbc.2023.105328 (PMC10654029; doi:10.1016/j.jbc.2023.105328)

PDBePISA analysis of crystal packing contacts in MET crystal structures

MET Y1234E/Y1235E∙tepotinib (PDB-ID: 8AU3), SG: F222, dimer

**chain A:**

- αC-helix: **crystal packing contacts** by H-bonds involving I1118, N1123 & T1126 (PISA interface #1)
- A-loop: 13 out of 32 residues not defined by electron density, **no crystal packing contacts.**

**chain B:**

- αC-helix: **crystal packing contact** by H-bond involving N1123 (PISA interface #2)
- A-loop: 13 out of 32 residues not defined by electron density**, no crystal packing contacts.**

**
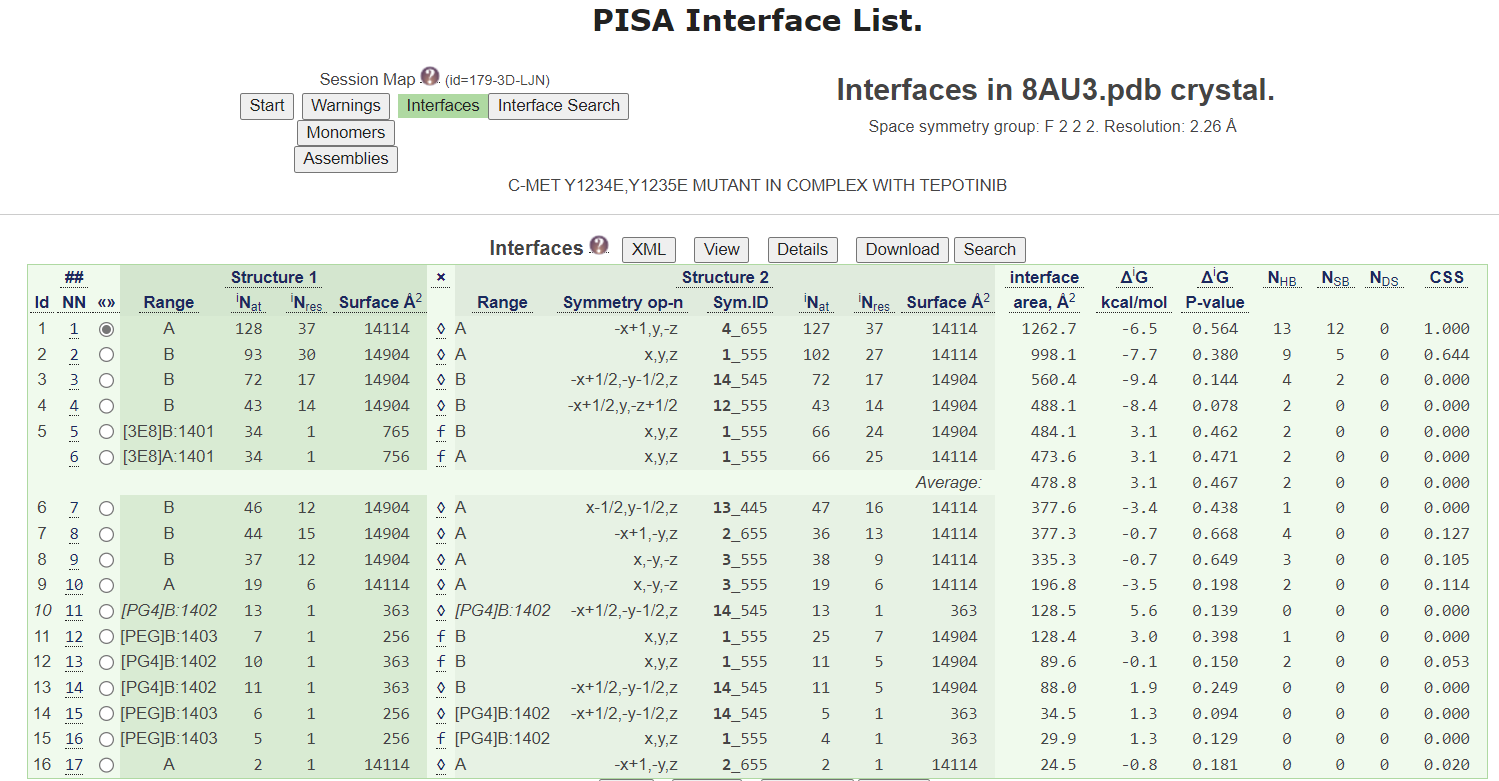
**

PISA interfaces with crystal packing contacts in MET Y1234E/Y1235E∙tepotinib (PDB-ID: 8AU3)

**
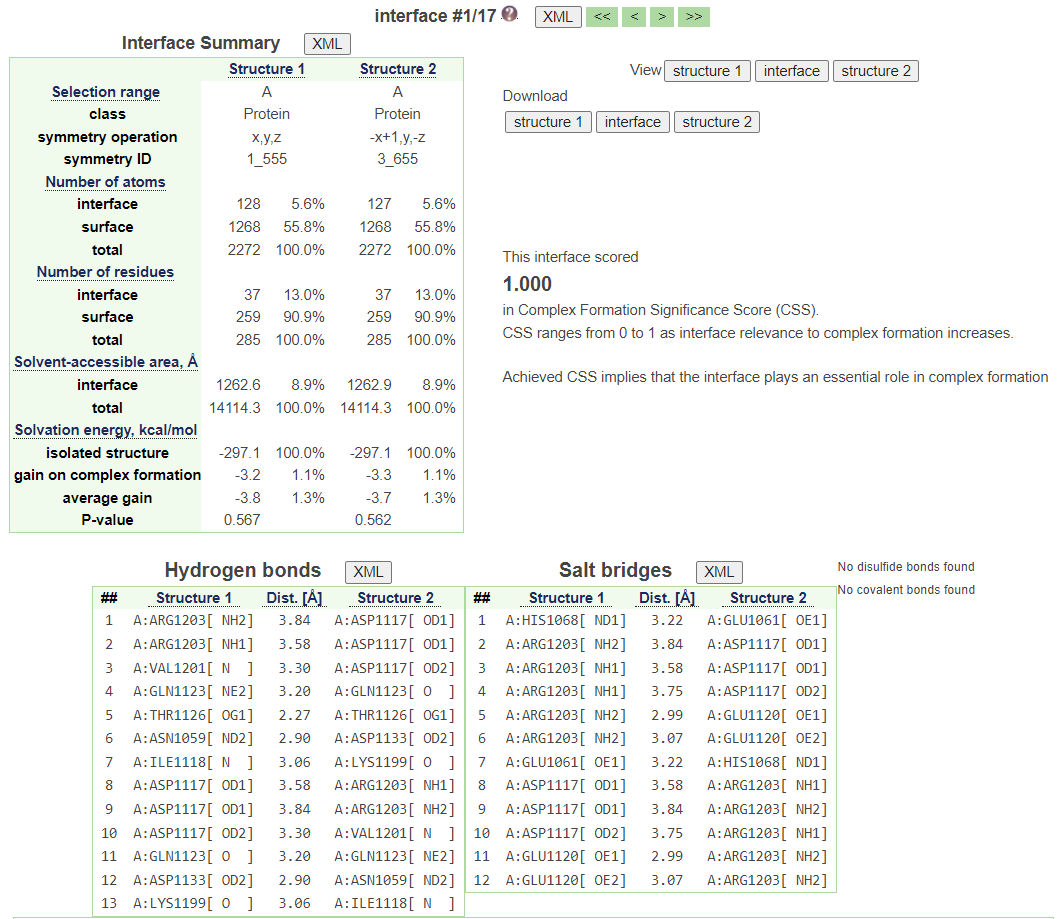
**

**
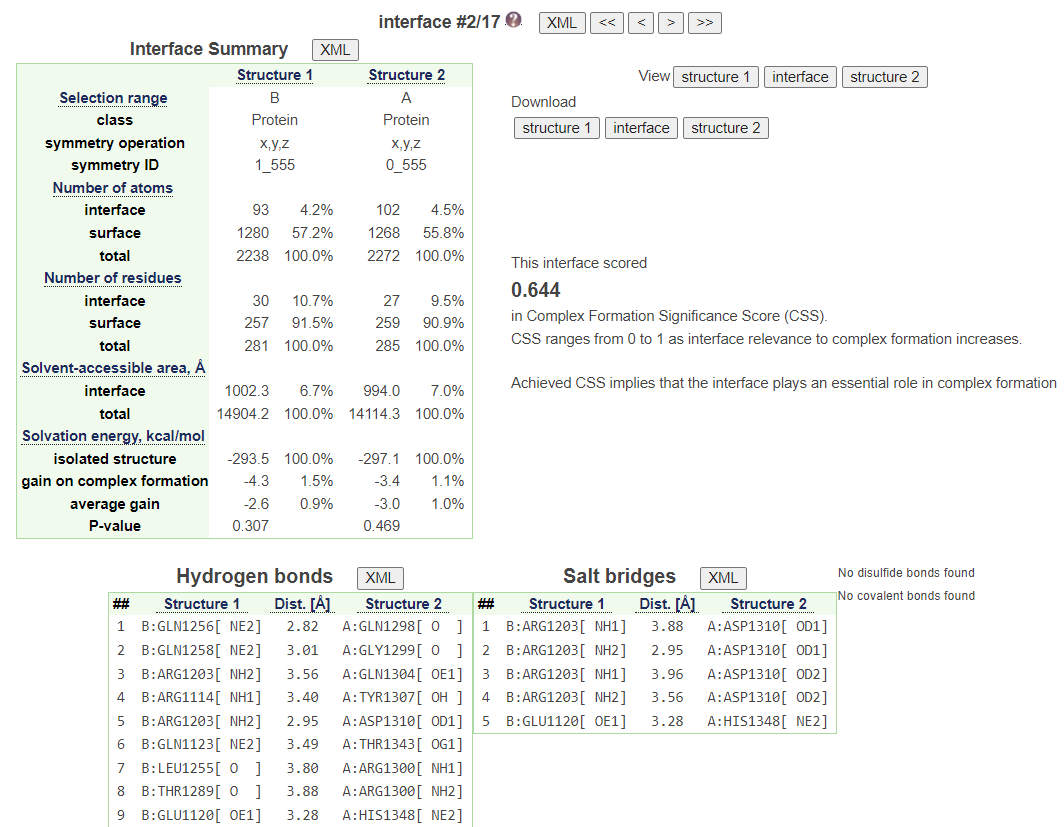
**

MET Y1235D∙tepotinib (PDB-ID: 8AW1), SG: P2_1_, dimer

**chain A:**

- αC-helix: residues 1114-1119 not defined. **crystal packing contact** by weak H-bond between S1122 (chain A) and E1082 (chain B) (PISA interface #5)
- A-loop: 8 (1236-1243) out of 32 residues not defined by electron density, **no crystal packing contacts.**

**chain B:**

- αC-helix: residues 1115-1118 not defined, **crystal packing contact** by weak H-bond between S1122 (chain A) and E1082 (chain B) (PISA interface #5)
- A-loop: 8 (1235-1242) out of 32 residues not defined by electron density, **no crystal packing contacts.**


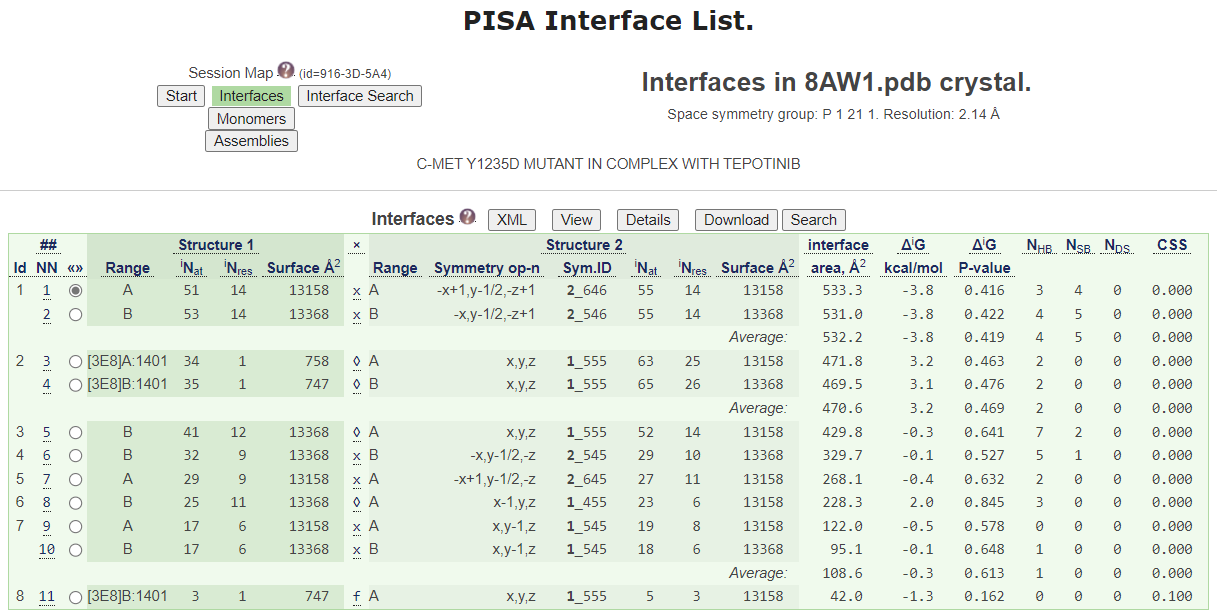


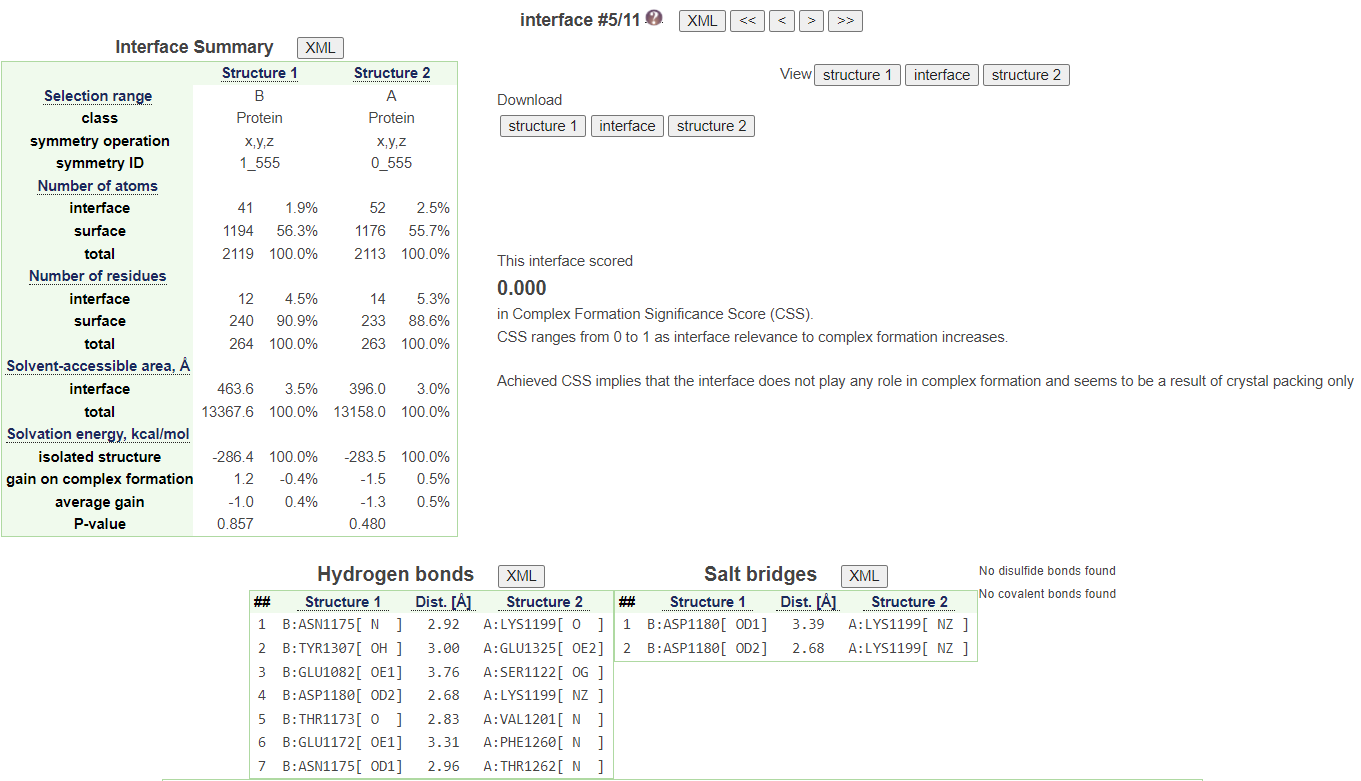


MET F1200I∙tepotinib (8AU5), SG: P2_1_, monomer

- αC-helix: residues 1115-1119 not defined, **crystal packing contact** by H-bond involving E1127 (PISA interface #3)
- A-loop: 2 (1238-1239) out of 32 residues not defined by electron density, **no crystal packing contacts.**


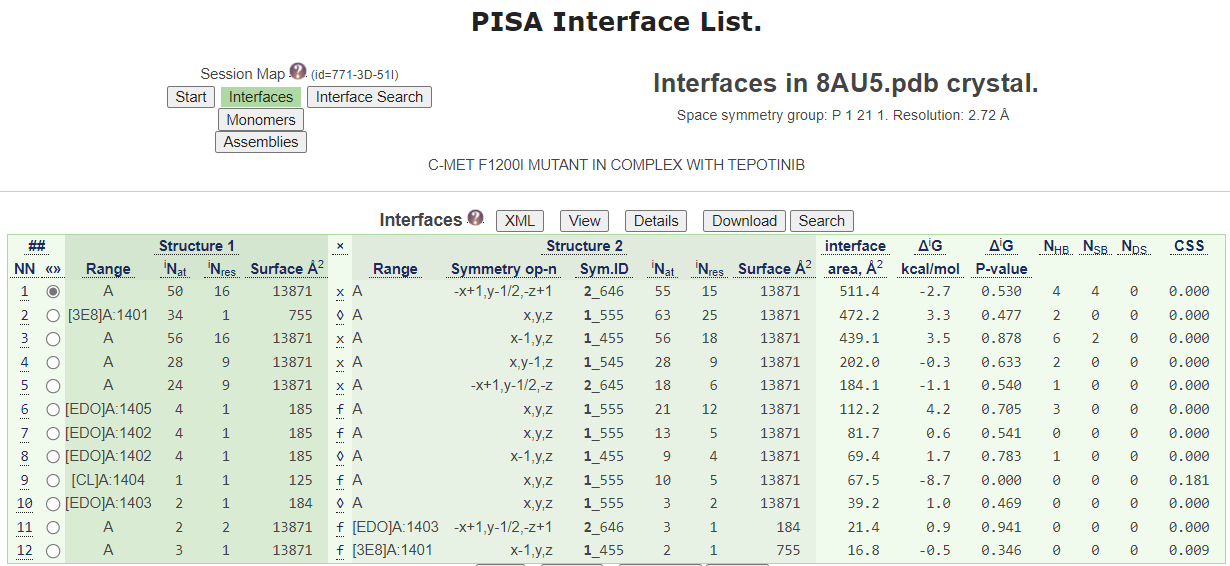


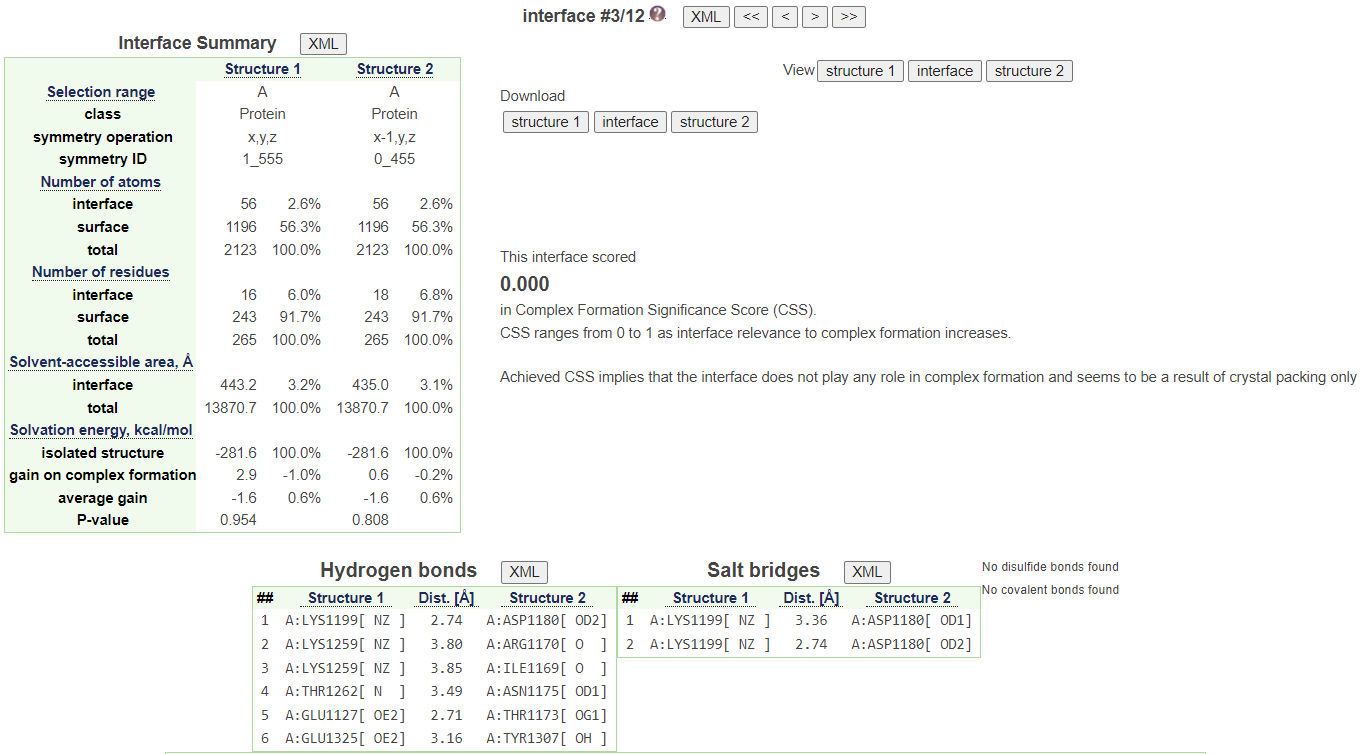


MET (wildtype)∙tepotinib (PDB-ID: 4R1V), SG: P2_1_, monomer

- αC-helix: **crystal packing contact** by H-bond involving D1133 (PISA interface #3)
- A-loop: all residues defined by electron density, **crystal packing contacts** by H-bonds involving K1232, E1233, N1239 (PISA interface #3)


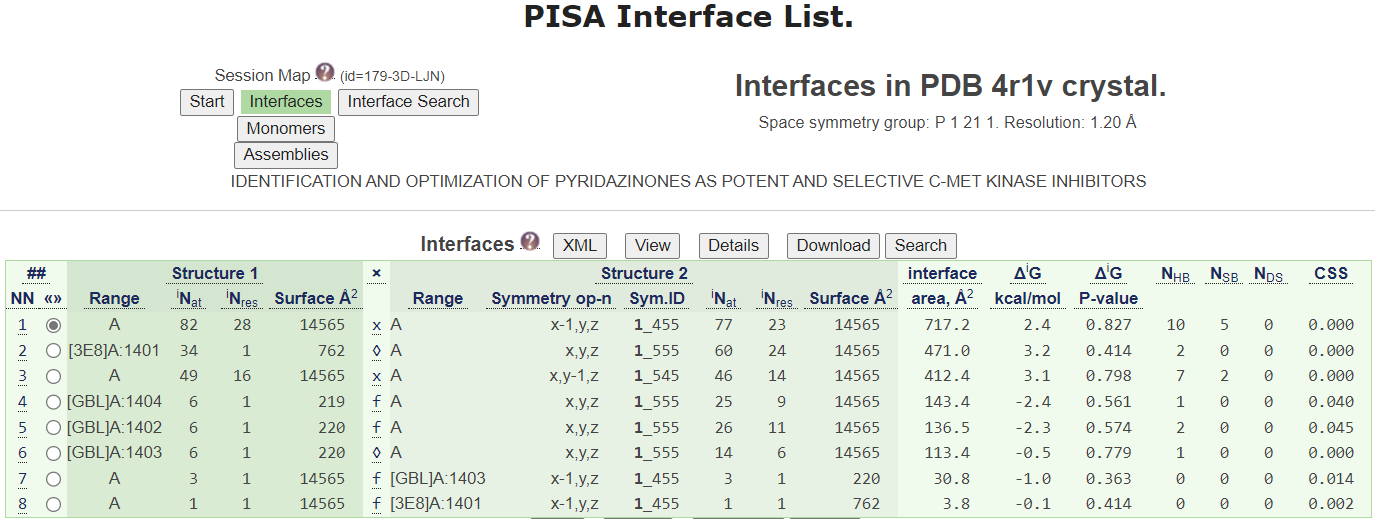


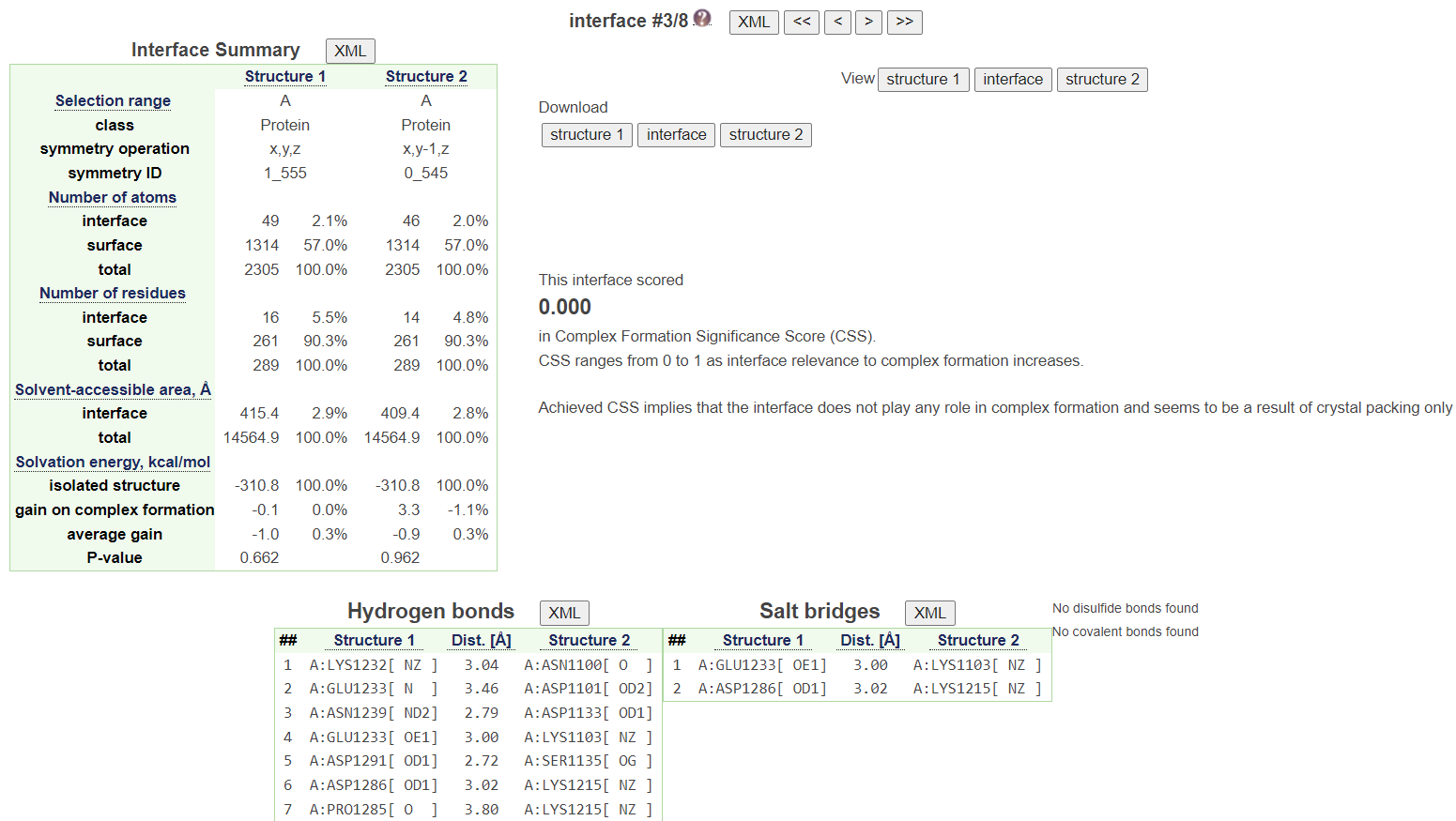


MET (wildtype, apo, pY1234/pY1235) (PDB-ID: 3Q6U), SG: P2_1_2_1_2_1_, monomer.

- αC-helix: **no crystal packing contact**.
- A-loop: 20 (1225-1244) out of 32 residues not defined by electron density, **no crystal packing contacts.**


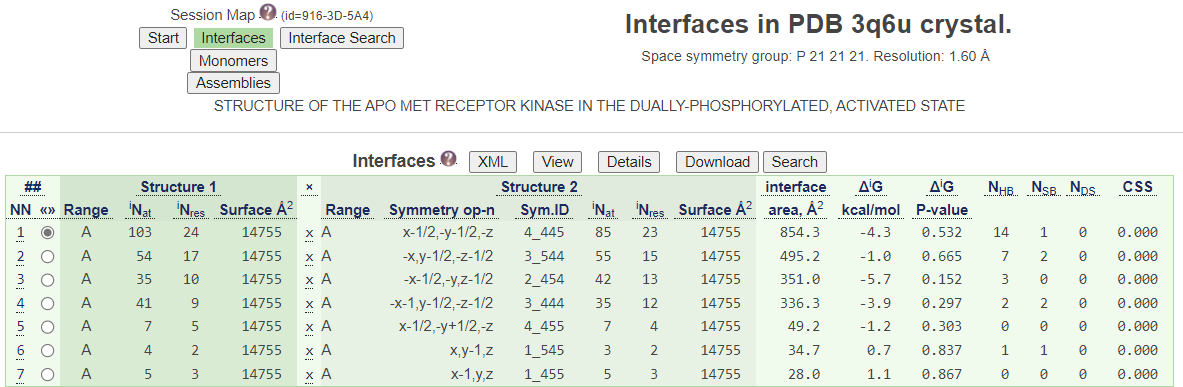


MET (wt, pY1234/pY1235) bound with type I inhibitor (PDB-ID: 3Q6W), SG: P2_1_2_1_2_1_, monomer

- αC-helix: **no crystal packing contact**
- A-loop: 4 (1240-1243) out of 32 residues not defined by electron density, **no crystal packing contacts**


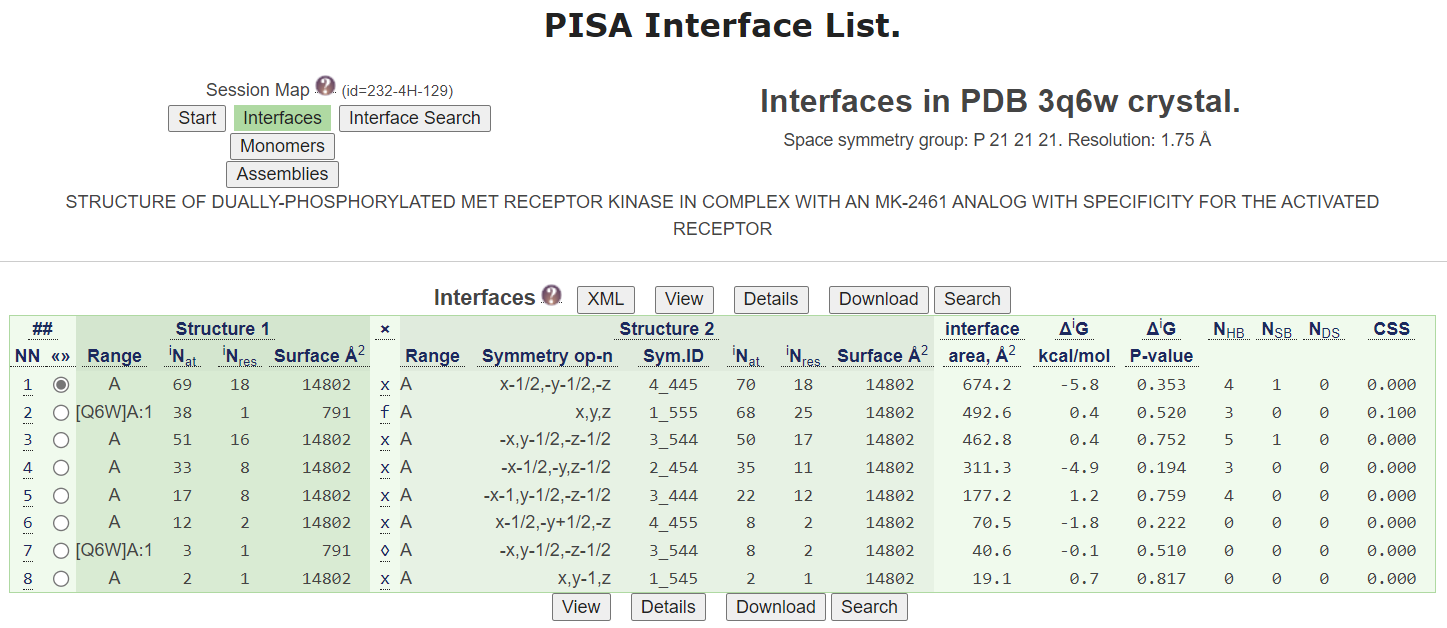

Supplement: Supporting Information 1 [file mmc2.docx]
