# Supplementary material for: Biophysical and structural characterization of the impacts of MET phosphorylation on tepotinib binding
Source: J Biol Chem. 2023 Oct 6;299(11):105328. doi: 10.1016/j.jbc.2023.105328 (PMC10654029; doi:10.1016/j.jbc.2023.105328)
Supplement: Supporting Information 2 [file mmc3.pdf]

PDB ID	SPACE GROUP	KinCore SpatialLabel	KinCore C-helix	Kinametrix Conformation Classification	Disordered residues in A-loop (chain A)	Ligand type	pi-stacking to Tyr1230	Mutations or phosphorylations	Crystal Growth Procedure	DOI
7b3q	C121	DFGin	out		7	Type1	no	None	25 % PEG3350, 0.2 M (NH4)2SO4, 0.1 M Na-HEPES pH 7.5	10.1021/acsmedchemlett.0c00392
7b44	C121	DFGin	out		7	Type1	no	None	25 % PEG3350, 0.2 M (NH4)2SO4, 0.1 M Na-HEPES pH 7.5	10.1021/acsmedchemlett.0c00392
7b3t	C121	DFGin	out		8	Type1	no	None	25 % PEG8000, 0.2 M Li2SO4	10.1021/acsmedchemlett.0c00392
7b41	C121	DFGin	out		8	Type1	no	None	25 % PEG3350, 0.2 M MgCl2, 0.1 M bis-tris pH 5.5	10.1021/acsmedchemlett.0c00392
7b42	C121	DFGin	out		8	Type1	no	None	25 % PEG3350, 0.2 M MgCl2, 0.1 M bis-tris pH 6.5	10.1021/acsmedchemlett.0c00392
7b3z	C121	DFGin	out		10	Type1	no	None	20 % PEG10000, 0.1 M Na-HEPES pH 7.5	10.1021/acsmedchemlett.0c00392
7b40	C121	DFGin	out		10	Type1	no	None	20 % PEG4000, 10 % isopropanol, 0.1 M Na-HEPES pH 7.5	10.1021/acsmedchemlett.0c00392
3a4p	C121	DFGin	out	cHelix out/DFG in	10	Type1	yes	Y1194F Y1234F Y1235D	14%(w/v) PEG MME 5000, 5%(v/v) isopropanol, 12%(v/v) MPD, 0.1M Tris-Cl, 15%(v/v) Glycerol, pH 7.5	10.1016/j.bmcl.2009.12.109
7b3v	C121	DFGin	out		14	Type1	no	None	25 % PEG3350, 0.2 M (NH4)2SO4, PCPT pH 5.5	10.1021/acsmedchemlett.0c00392
3u6i	C2221	DFGin	out	cHelix out/DFG in	20	Type1.5_Front	no	None	12% PEG 6000, 1.0 M lithium chloride, 0.1 M sodium citrate, pH 5.0	10.1021/jm201330u
3u6h	C2221	DFGin	out	cHelix out/DFG in	21	Type1.5_Front	no	None	12% PEG 6000, 1.0 M lithium chloride, 0.1 M sodium citrate, pH 5.0	10.1021/jm201330u
5t3q	C2221	None	out		22	Type1.5_Front	no	None	12% PEG 6000, 1.0 M lithium chloride, 0.1 M sodium citrate (pH 5.0	unpublished
8au3	F222	DFGin	out	cHelix out/DFG in	13	tepotinib	yes	Y1234E Y1235E	0.10 M MES pH=6.50, 3.4 M sodium formiate	this paper
3f66	P1	DFGin	out	cHelix out/DFG in	5	Type1	no	None		10.1016/j.bmcl.2008.11.062
3qti	P1	DFGin	out	cHelix out/DFG in	10	Type1	yes	Y1194F Y1234F Y1235D	100 mM Hepes pH 7.5, 16% PEG 4000, 8% isopropanol, and 3 mM TCEP	10.1158/0008-5472.CAN-10-4433
7b43	P1	DFGin	out		10	Type1	yes	None	28 % PEGMME2000, 0.1 M bis-tris pH 6.5	10.1021/acsmedchemlett.0c00392
5hni	P1	DFGin	out	cHelix out/DFG in	18	Type1	yes	None	Hepes 100mM, isopropanol 11%, PEG5000 MME 6%	10.1021/acs.jmedchem.6b00280
3rhk	P1	DFGout	out	cHelix out/DFG out	0	Type1	no	None	13% ethanol, 12% ethylene glycol, 100mM imidazole, pH 8.5, HANGING DROP, temperature 277K	10.1074/jbc.M110.213801
8an8	P1	DFGout	out		10	Type1.5_Front	no	None	0.4 M am sulfate, PCPT pH 5.5	10.1016/j.bmcl.2022.128948
4r1v	P1211	DFGin	out	cHelix out/DFG in	0	tepotinib	yes	None	PEG 8000, pH 6.5, temperature 298K	10.1016/j.bmcl.2015.02.002
8au5	P1211	DFGin	out	cHelix out/DFG in	2	tepotinib	yes	F1200I	8%-18% (w/v) PEG-4000, 11%-17% (v/v) isopropanol) at 293K	this paper
8aw1	P1211	DFGin	out	cHelix out/DFG in	8	tepotinib	yes	Y1235D	8%-18% (w/v) PEG-4000, 11%-17% (v/v) isopropanol) at 293K	this paper
2wd1	P1211	DFGin	out	cHelix out/DFG in	0	Type1	yes	None		10.1016/J.BMCL.2009.03.110
4knb	P1211	DFGin	out	cHelix out/DFG in	0	Type1	yes	None		10.1016/j.bmcl.2013.05.074
5eob	P1211	DFGin	out	cHelix out/DFG in	3	Type1	yes	None	19-20%(w/v)PEG 3350, 200mM MgSO4, 100mM Tris-HCl	10.1016/j.ejmech.2016.03.076
5dg5	P1211	DFGout	in	cHelix in/DFG out	0	Type2	no	None	1.0M diammonium hydrogen phosphate, 0.2M sodium chloride, 0.1M citrate pH 5.0 and 7.5% glycero	10.1158/1535-7163.MCT-14-1105
3lq8	P1211	DFGout	in	cHelix out/DFG out	6	Type2	no	None	12% PEG 4000, 15% isopropanol, 25 mM MOPS, pH 6.5, 150 mM NaCl, 2 mM DTT	10.1158/0008-5472.CAN-08-4889
2wgj	P21212	DFGin	out	cHelix out/DFG in	0	Type1	yes	None	0-275 MM SODIUM CHLORIDE, 21% (W/V PEG 3350, 50 MM CITRATE-PHOSPHATE PH 4.6	10.1021/JM2007613
2wkm	P21212	DFGin	out	cHelix out/DFG in	0	Type1	yes	None	0.05 M CITRATE-PHOSHPHATE 4.6, 0-0.275 M NACL, AND 21% W/V PEG 3350	10.1021/JM2007613
3zbx	P21212	DFGin	out	cHelix out/DFG in	0	Type1	yes	None	0.05M CITRATE-PHOSPHATE PH 4.2, 200M NACL, AND 17.4% PEG3,350	10.1021/JM400926X
3zc5	P21212	DFGin	out	cHelix out/DFG in	0	Type1	yes	None	0.05 M CITRATE-PHOSPHATE PH 4.6, 0-0.275 M NACL, AND 17-21% PEG3,350	10.1021/JM400926X
3zcl	P21212	DFGin	out	cHelix out/DFG in	0	Type1	yes	None	0.05 M CITRATE-PHOSPHATE PH 4.6, 0-0.275 M NACL, AND 17-21% PEG3,350	10.1021/JM400926X
3zxz	P21212	DFGin	out	cHelix out/DFG in	0	Type1	yes	None	0.05 M CITRATE-PHOSHPHATE 4.6, 0-0.275 M NACL, AND 21% W/V PEG 3350	10.1021/JM300967G
3zze	P21212	DFGin	out	cHelix out/DFG in	0	Type1	yes	None	0.05 M CITRATE-PHOSHPHATE 4.6, 0-0.275 M NACL, AND 21% W/V PEG 3350	10.1021/JM300967G
4aoi	P21212	DFGin	out	cHelix out/DFG in	0	Type1	yes	None	0.05 M CITRATE-PHOSPHATE, PH 4.6, 0-25 MM NACL, 21 % (W/V) PEG-3350	10.1021/JM300967G
4ap7	P21212	DFGin	out	cHelix out/DFG in	0	Type1	yes	None	0.05 M CITRATE-PHOSPHATE, PH 4.6, 0-25 MM NACL, 21 % (W/V) PEG-3350	10.1021/JM300967G
3q6u	P212121	DFGin	in	cHelix in/DFG in	20	No_ligand	no	pY1234 pY1235	150 mM malic acid, 20% PEG3350., pH 7.0	10.1074/jbc.M110.204404
3q6w	P212121	DFGin	in	cHelix in/DFG in	4	Type1	no	pY1234 pY1235	150 mM malic acid, 20% PEG3350	10.1074/jbc.M110.204404
3r7o	P212121	DFGin	in	cHelix in/DFG in	4	Type1	no	pY1234 pY1235	150 mM malic acid, 20% PEG3350, pH 7.0	10.1074/jbc.M110.204404
4iwd	P212121	DFGin	in	cHelix in/DFG in	4	Type1	no	pY1234 pY1235	150 mM malic acid, 20% PEG3350, pH 7.0	10.1021/jm301619u
3dkc	P212121	DFGin	out	cHelix out/DFG in	0	ATP	no	Y1194F Y1234F Y1235D	11% ISOPROPANOL, 2.5% PEG 5K MME, 100 mM BIS-TRIS, pH 6.2, temperature 292K	10.1158/1535-7163.MCT-09-0477
1r1w	P212121	DFGin	out	cHelix out/DFG in	14	No_ligand	no	Y1194F Y1234F Y1235D L1272L	PEG 5000 MME, isopropanol, Hepes, pH 7.10	10.1073/pnas.1734128100
1r0p	P212121	DFGin	out		0	Type1	yes	Y1194F Y1234F Y1235D L1272L	PEG 5000 MME, isopropanol, Hepes, pH 7.1	10.1073/pnas.1734128100
4deg	P212121	DFGin	out	cHelix out/DFG in	0	Type1	yes	None	15% PEG 4000, 0.1 M HEPES, 6% isopropanol, 3% ethanol, 40 mM beta-mercaptoethanol, pH 7.8	10.1016/j.bmcl.2012.04.072
4deh	P212121	DFGin	out	cHelix out/DFG in	0	Type1	yes	None	15% PEG 4000, 0.1 M HEPES, 6% isopropanol, 3% ethanol, 40 mM beta-mercaptoethanol, pH 7.8	10.1016/j.bmcl.2012.04.072
4dei	P212121	DFGin	out	cHelix out/DFG in	0	Type1	yes	None	15% PEG 4000, 0.1 M HEPES, 6% isopropanol, 3% ethanol, 40 mM beta-mercaptoethanol, pH 7.8	10.1016/j.bmcl.2012.04.072
4xmo	P212121	DFGin	out	cHelix out/DFG in	0	Type1	yes	None	12% PEG 4000, 100 mM HEPES, pH 7.8, 6% isopropanol, 3% ethanol, 40 mM beta-mercaptoethano	10.1021/jm501913a
4xyf	P212121	DFGin	out	cHelix out/DFG in	0	Type1	yes	None	13% PEG 4000, 100 mM HEPES, 6% (v/v) isopropanol, 3% (v/v) ethanol, 40 mM beta-mercaptoethano	10.1021/jm501913a
5eyc	P212121	DFGin	out	cHelix out/DFG in	0	Type1	yes	None	12% PEG 4000, 3% (v/v) ethanol, 6% (v/v) isopropanol, 40 mM beta-mercaptoethanol, 100 mM HEPES (pH 7.8	10.1021/acs.jmedchem.5b01716
5eyd	P212121	DFGin	out	cHelix out/DFG in	0	Type1	yes	None	12% PEG 4000, 3% (v/v) ethanol, 6% (v/v) isopropanol, 40 mM beta-mercaptoethanol, 100 mM HEPES (pH 7.8	10.1021/acs.jmedchem.5b01716
5uab	P212121	DFGin	out		0	Type1	yes	None	16.% PEG MME 5000, 15.% Isopropanol, 0.1M HEPES pH 7.8	10.1158/1535-7163.MCT-16-0771
5uad	P212121	DFGin	out		0	Type1	yes	None	19.% PEG 3350, 12.% Isopropanol, 0.1M HEPES pH 7.2	10.1158/1535-7163.MCT-16-0771
5ya5	P212121	DFGin	out		0	Type1	yes	None	0.1M Tris pH7.5, 15% glycerol, 12% MPD, 5% isopropanol, 15% PEG5Kmme	10.1016/j.ejmech.2017.11.073
6sde	P212121	DFGin	out		0	Type1	yes	None	15 % 2-propanol, 15 % PEG4K, 0.2 M PCPT pH 7.5	10.1021/acsmedchemlett.9b00276
6ubw	P212121	DFGin	out		0	Type1	yes	None	12% isopropanol, 10% PEG5000 MME, 0.06 M HEPES sodium, 0.04 M HEPES	10.1158/1535-7163.MCT-16-0771
3ccn	P212121	DFGin	out	cHelix out/DFG in	1	Type1	yes	None	0.1M HEPES pH 7.8, 15% PEG 4K, 6% 2-Propanol, 40mM BME, and 3% Ethanol	10.1021/jm800043g
3cd8	P212121	DFGin	out	cHelix out/DFG in	1	Type1	yes	None	0.1M HEPES pH 7.8, 15% PEG 4K, 6% 2-Propanol, 40mM BME, and 3% Ethano	10.1021/jm800043g
2rfs	P212121	DFGin	out	cHelix out/DFG in	2	Type1	yes	None	0.1M HEPES pH 7.8, 15% PEG 4K, 6% 2-propanol, 40mM BME, 3% Ethanol, temperature 298K	10.1074/jbc.M705774200

5hlw	P212121	DFGin	out	cHelix out/DFG in	5	Type1	no	Y1230H	Tris 100mM-MPD20%-pH8	10.1021/acs.jmedchem.6b00280
3i5n	P212121	DFGin	out	cHelix out/DFG in	5	Type1	yes	None	15% PEG 4000, 0.1 M HEPES, 40 mM beta-mercaptoethanol, 6% isopropanol, 3% ethanol, pH 7.8	10.1016/j.bmcl.2009.09.096
3dkf	P212121	DFGin	out	cHelix out/DFG in	13	Type1	no	Y1194F Y1234F Y1235D	20% ISOPROPANOL, 200 mM AMMONIUM ACETATE, 100 mM TRIS, pH 7.5, temperature 292K	10.1158/1535-7163.MCT-09-0477
5hor	P212121	DFGin	out	cHelix out/DFG in	15	Type1	no	Y1194F Y1234F Y1235D M1250T	Tris 100mM, MPD 20%, pH8.5	10.1021/acs.jmedchem.6b00280
3dkg	P212121	DFGin	out	cHelix out/DFG in	17	Type1	no	Y1194F Y1230L Y1234F Y1235D	11% ISOPROPANOL, 3% PEG 5K MME, 100 mM BIS-TRIS, pH 5.8, temperature 292K	10.1158/1535-7163.MCT-09-0477
5ho6	P212121	DFGin	out	cHelix out/DFG in	17	Type1	no	Y1194F Y1234F Y1235D	Tris 100mM - MPD 20% - pH8.5	10.1021/acs.jmedchem.6b00280
5hoa	P212121	DFGin	out	cHelix out/DFG in	17	Type1	no	None	Tris 100 mM, MPD 24%, pH8.5	10.1021/acs.jmedchem.6b00280
3efk	P212121	DFGin	out	cHelix out/DFG in	22	Type1.5_Front	no	L1272L	12% PEG 6000, 1.0M LiCl2, 0.1M Sodium Citrate, pH 5.0	10.1021/jm8006189
4mxc	P212121	DFGout	in	cHelix out/DFG out	5	Type2	no	None	19-20%(w/v)PEG 3350, 200mM MgSO4, 100mM Tris-HCl, pH 7.5	10.1021/ml500066m
5hti	P212121	DFGout	in		5	Type2	no	None	10-11% PEG5000MME, 11% isopropanol, 0.1M HEPES pH 7.1	unpublished
6sd9	P212121	DFGout	in		5	Type2	no	None	15 % 2-propanol, 17 % PEG4K, 0.1 M NaHEPES pH 8	10.1021/acsmedchemlett.9b00276
6sdc	P212121	DFGout	in		5	Type2	no	D1228V	15 % 2-propanol, 10 % PEG4K, 0.1 M NaHEPES pH 8	10.1021/acsmedchemlett.9b00276
3vw8	P212121	DFGout	in	cHelix out/DFG out	6	Type2	no	None	0.1M HEPES, 25% PEG2000, 8% isopropanol, pH 7.5	10.1016/j.bmc.2013.10.028
7v3s	P212121	DFGout	in		7	Type2	no	None	0.1M HEPES, 8% isopropanol, 3mM TECP, 16% PEG4000, pH 7.5	10.1021/acs.jmedchem.2c00962
7v3r	P212121	DFGout	in		8	Type2	no	None	0.1 M HEPES, 8% isopropanol, 3 mM TECP, 16% PEG4000, pH7.5	10.1021/acs.jmedchem.2c00962
3l8v	P212121	DFGout	in	cHelix in/DFG out	14	Type2	no	Y1194F Y1234F Y1235D		10.1016/j.bmcl.2010.01.042
4eev	P212121	DFGout	in	cHelix in/DFG out	19	Type2	no	None	16% PEG 10.000, 0.1 M HEPES, and 5% ethylene glycol, pH 7.0	10.1007/s10637-012-9912-9
3c1x	P212121	DFGout	in	cHelix in/DFG out	21	Type2	no	Y1194F Y1234F Y1235D L1272L		10.1016/j.bmcl.2008.01.121
3ctj	P212121	DFGout	in	cHelix in/DFG out	21	Type2	no	Y1194F Y1234F Y1235D	12% MEPEG 5000, 0.1M HEPES (PH 7.1) 11% 2-PROPANOL	10.1016/j.bmcl.2008.04.047
8ans	P212121	DFGout	out		15	Type1.5_Front	no	D1228V	15 % PEG10K, 100 mM PCPT pH 7.5	10.1016/j.bmcl.2022.128948
3efj	P212121	DFGout	out	cHelix out/DFG out	22	Type1.5_Front	no	L1272L	12% PEG 6000, 1.0M LiCl2, 0.1M Sodium Citrate, pH 5.0	10.1021/jm8006189
6sdd	P212121	DFGout	out		0	Type2	no	D1228V	8 % ethanol, 20 % PEG8K, 0.1 M PCPT pH 7.5	10.1021/acsmedchemlett.9b00276
3ce3	P212121	DFGout	out	cHelix out/DFG out	14	Type2	no	Y1194F Y1234F Y1235D L1272L	12% MEPEG 5000, 0.1M HEPES, 11% 2-PROPANOL., pH 7.1	10.1021/jm800476q
3cth	P212121	DFGout	out	cHelix in/DFG out	14	Type2	no	Y1194F Y1234F Y1235D	12% MEPEG 5000, 0.1M HEPES (PH 7.1) 11% 2-PROPANOL	10.1016/j.bmcl.2008.04.047
3f82	P212121	DFGout	out	cHelix in/DFG out	19	Type2	no	Y1194F Y1234F Y1235D	12% MEPEG 5000, 0.1M HEPES (PH 7.1) 11% 2-PROPANOL	10.1021/jm801586s
2rfn	P212121	None	out		23	Type1.5_Front	no	None	12% PEG 6000, 1.0M LiCl2, 0.1M sodium citrate, pH 5.0, temperature 298K	10.1074/jbc.M705774200
2g15	P213	DFGin	out		0	No_ligand	no	None	1 M diammonium hydrogen phosphate, 0.2 M Sodium chloride, 0.1 M citrate(pH 5.0), and 7.5% glycero	10.1073/pnas.0600048103
7b3w	P41212	DFGin	None		0	Type1	yes	None	8 % ethylene glycol, 10 % PEG8000, 0.1 M Na-HEPES pH 7.5	10.1021/acsmedchemlett.0c00392
4gg5	P41212	DFGin	out	cHelix out/DFG in	0	Type1	yes	None	0.1M HEPES, 8% isopropanol, 3mM TECP, 16% PEG4000, pH 7.5	10.1016/j.bmcl.2012.08.075
4gg7	P41212	DFGin	out	cHelix out/DFG in	0	Type1	yes	None	0.1M Tris-HCl, 15% glycerol, 12% MPD, 5% isopropanol, 14% PEG5000, pH 7.5	10.1016/j.bmcl.2012.08.075
7y4t	P41212	DFGin	out		0	Type1	yes	None	0.1 M HEPES (pH 7.8), 15-30% (v/v) PEG 8000	10.1021/acs.jmedchem.2c00981
7y4u	P41212	DFGin	out		0	Type1	yes	None	0.1 M HEPES (pH 7.8), 15-30% (v/v) PEG 8000	10.1021/acs.jmedchem.2c00981
8gvj	P41212	DFGin	out		0	Type1	yes	None	25% PEG 8K	10.1021/acs.jmedchem.2c00981
